# Dynamic shifts in occupancy by TAL1 are guided by GATA factors and drive large-scale reprogramming of gene expression during hematopoiesis

**DOI:** 10.1101/gr.164830.113

**Published:** 2014-12

**Authors:** Weisheng Wu, Christapher S. Morrissey, Cheryl A. Keller, Tejaswini Mishra, Maxim Pimkin, Gerd A. Blobel, Mitchell J. Weiss, Ross C. Hardison

**Affiliations:** 1 Center for Comparative Genomics and Bioinformatics, Department of Biochemistry and Molecular Biology, The Pennsylvania State University, University Park, Pennsylvania 16802, USA;; 2 Division of Hematology, The Children’s Hospital of Philadelphia, Philadelphia, Pennsylvania 19104, USA;; 3 Perelman School of Medicine at the University of Pennsylvania, Philadelphia, Pennsylvania 19104, USA

## Abstract

We used mouse ENCODE data along with complementary data from other laboratories to study the dynamics of occupancy and the role in gene regulation of the transcription factor TAL1, a critical regulator of hematopoiesis, at multiple stages of hematopoietic differentiation. We combined ChIP-seq and RNA-seq data in six mouse cell types representing a progression from multilineage precursors to differentiated erythroblasts and megakaryocytes. We found that sites of occupancy shift dramatically during commitment to the erythroid lineage, vary further during terminal maturation, and are strongly associated with changes in gene expression. In multilineage progenitors, the likely target genes are enriched for hematopoietic growth and functions associated with the mature cells of specific daughter lineages (such as megakaryocytes). In contrast, target genes in erythroblasts are specifically enriched for red cell functions. Furthermore, shifts in TAL1 occupancy during erythroid differentiation are associated with gene repression (dissociation) and induction (co-occupancy with GATA1). Based on both enrichment for transcription factor binding site motifs and co-occupancy determined by ChIP-seq, recruitment by GATA transcription factors appears to be a stronger determinant of TAL1 binding to chromatin than the canonical E-box binding site motif. Studies of additional proteins lead to the model that TAL1 regulates expression after being directed to a distinct subset of genomic binding sites in each cell type via its association with different complexes containing master regulators such as GATA2, ERG, and RUNX1 in multilineage cells and the lineage-specific master regulator GATA1 in erythroblasts.

Dynamic changes in the locations and actions of transcription factors (TFs) are thought to drive much of the differential gene expression that determines cell fate, morphology, and function (Davidson and Erwin 2006). Recent genome-wide determinations of TF occupancy in multiple stages of hematopoiesis (Kassouf et al. 2010; Wilson et al. 2010), coupled with new data from the Mouse ENCODE Project (Wu et al. 2011; The Mouse ENCODE Consortium et al. 2012; The Mouse ENCODE Consortium et al. 2014; Pimkin et al. 2014), allow us to examine in detail the patterns of differential occupancy by key TFs during hematopoietic differentiation, correlate this dynamic binding with changes in gene expression, and search for determinants of differential occupancy.

Here we focused on TAL1 (previously known as SCL), a TF that is indispensable at multiple stages of hematopoiesis. This basic helix-loop-helix (bHLH) protein is required to establish hematopoietic stem cells during embryogenesis and also to differentiate along the erythroid and multiple myeloid cell lineages, including those leading to megakaryocytes, mast cells, and eosinophils. The requirement for TAL1 in these processes has been demonstrated by multiple in vivo and in vitro genetic experiments. Homozygous *Tal1*-null murine embryos die of anemia with failed yolk sac hematopoiesis (Robb et al. 1995; Shivdasani et al. 1995). Furthermore, no hematopoietic lineages were detectable from *Tal1*-null embryonic stem cells after in vitro differentiation or in chimeric mice (Porcher et al. 1996). Conditional *Tal1* knockout and rescue experiments show that TAL1 is also needed for specification and differentiation of erythroid and megakaryocytic cells (Schlaeger et al. 2005). TAL1 is expressed broadly in erythropoiesis, from highly proliferative, committed progenitor cells (BFU-e and CFU-e) to more mature erythroblasts (Aplan et al. 1992; Porcher et al. 1996). In contrast, TAL1 is normally absent from lymphoid cells, but its aberrant expression in T cells leads to T-cell acute lymphocytic leukemia (Palii et al. 2011). The pleotropic effects of *Tal1* mutations in hematopoietic stem cells and in multiple hematopoietic lineages suggest that the TAL1 protein plays unique roles in each stage and lineage. These roles could be realized in either or both of two ways: by binding to different locations in the genome to regulate distinct sets of genes in each cell type, and by interacting with different proteins to carry out distinct functions, such as activation or repression.

One determinant of TAL1 binding to DNA is the sequence preference of its DNA-binding domain. Binding-site selection experiments in solution have shown that TAL1, as a heterodimer with other bHLH proteins such as the E-protein TCF3 (E47) (Hsu et al. 1994), binds to the consensus sequence AACAGATGGT, which contains a subset of E-box motifs (CANNTG) (Church et al. 1985). Other studies showed preferential binding to CAGGTG (Wadman et al. 1997) and CAGCTG (Kassouf et al. 2010), implying that CAGVTG is the preferred consensus sequence. Remarkably, the DNA binding domain is not required for all TAL1 functions. Mutant ES cells homozygous for an intrinsic DNA-binding-domain–defective *Tal1* allele (*Tal1*^*rer*^) still support primitive erythropoiesis (Porcher et al. 1999), and mouse embryos homozygous for this mutation survive past 9.5 dpc, when the *Tal1* homozygous null mice die (Kassouf et al. 2008). These results show that direct binding to DNA is dispensable for some TAL1 functions in primitive erythropoiesis. Furthermore, a motif search on TAL1 binding sites in human proerythroblasts revealed that E-boxes are absent from over one-fifth of the sites. Indeed, GATA motifs ranked as the most overrepresented motifs, and they were closer to TAL1 peak summits than E-boxes (Tripic et al. 2009; Palii et al. 2011). Another study compared TAL1 binding sites in primary erythroid progenitor cells from wild-type mice and from *Tal1*^*rer/rer*^ mice (lacking the TAL1 DNA binding domain) and found that one-fifth of the wild-type TAL1 binding sites were also occupied in the mutant mice (Kassouf et al. 2010). This ability of DNA-binding-domain–defective TAL1 to bind specific genomic locations suggests that it may be recruited by other DNA-binding TFs.

Some of the TAL1 in the nucleus is in a multiprotein complex with the TFs GATA1 (or GATA2), LMO2, and LDB1; this complex binds to specific *cis*-regulatory elements in erythroid cells (Wadman et al. 1997; Anguita et al. 2004; Schuh et al. 2005; Cheng et al. 2009). In the hematopoietic precursor cell line HPC-7, which exhibits multilineage myeloid and erythroid potential (Pinto do O et al. 1998), additional TFs, including LYL1, RUNX1, ERG, and FLI1, coassociate with the bound TAL1-containing complex (Wilson et al. 2010). Cobinding of different TFs with TAL1 affects its function. When bound together with GATA1, TAL1 is strongly associated with activation of gene expression in erythroid cells. In multiple models for erythroid differentiation, a substantial majority of induced genes are co-occupied by both GATA1 and TAL1, whereas a subset of GATA1-repressed genes is bound by GATA1 but not TAL1 (Wozniak et al. 2008; Cheng et al. 2009; Tripic et al. 2009; Soler et al. 2010; Wu et al. 2011). Furthermore, the activity of GATA1-occupied DNA segments (GATA1 OSs) as enhancers is associated with co-occupancy by TAL1 (Tripic et al. 2009) and is dependent on an intact binding site (E-box) for TAL1 (Elnitski et al. 1997). In contrast, TAL1 binding to some genes operates as a molecular switch, leading to activation or repression under different conditions (Huang et al. 1999; Huang and Brandt 2000; Elnitski et al. 2001). These studies indicate that different cell-type–specific functions of TAL1 are regulated by the composition and activity of its interacting proteins.

The widely differing phenotypes of cells expressing active TAL1 predict that its regulated gene targets differ significantly. Consequently, the DNA segments occupied by this protein should differ between cell types. This prediction can now be evaluated comprehensively and quantitatively in mouse cell models. Recent studies from our laboratory, as part of the Mouse ENCODE Project (The Mouse ENCODE Consortium et al. 2014), and others have used chromatin immunoprecipitation (ChIP) followed by second-generation sequencing (ChIP-seq) (Johnson et al. 2007; Robertson et al. 2007) and related methods to map DNA segments occupied by TAL1 and other TFs across the genomes of multiple human and mouse hematopoietic cells of different lineages and at progressive stages of maturation (Cheng et al. 2009; Wilson et al. 2009, 2010; Kassouf et al. 2010; Soler et al. 2010; Palii et al. 2011; Tijssen et al. 2011; Wu et al. 2011; Dore et al. 2012; Kowalczyk et al. 2012; Xu et al. 2012; Pimkin et al. 2014). To gain further insights into the functions carried out by TAL1 in each cell type, we integrated these maps of TAL1 occupancy to establish its patterns of cell lineage–specific and maturational stage–specific occupancy and correlated these with gene expression. We studied the roles of histone modifications, matches to binding site motifs, and TF co-occupancy in determining differential TAL1 occupancy in different cell types. The results indicate that TAL1 is a potent regulator of hematopoiesis whose specificity is directed by other hematopoietic TFs.

## Results

### Substantial changes in occupancy by TAL1 during differentiation

**Figure 1.**
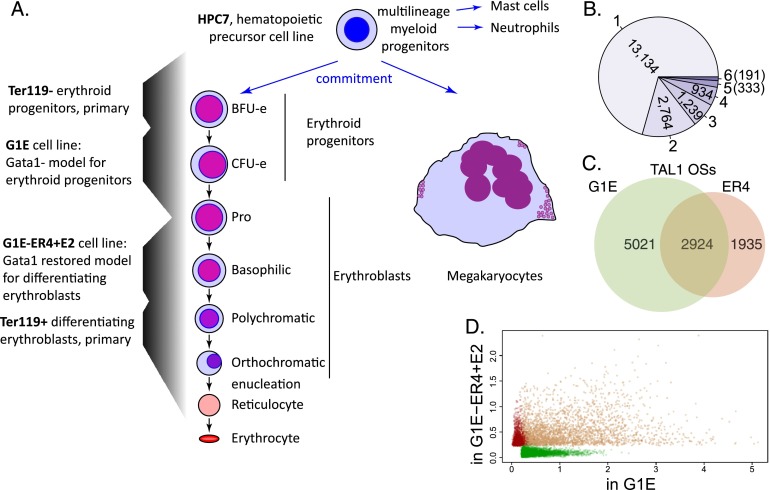
Erythropoiesis, megakaryopoiesis, and relocation of TAL1 occupancy. (*A*) The diagram shows differentiation from hematopoietic precursors to erythroblasts and megakaryocytes, including the corresponding cell types or lines used in this study. (*B*) The numbers of merged TAL1 OSs that are occupied by TAL1 in one to six of the assayed cell types or lines. (*C*) Venn diagram showing cell type–specific and shared TAL1 OSs in G1E and G1E-ER4 + E2 (ER4) cell lines. (*D*) Scatter plot showing the normalized ChIP-seq read counts of TAL1 on the TAL1 OSs in G1E versus ER4 cells. The TAL1 OSs identified only in G1E, only in ER4, or both are represented by green, red, or brown dots, respectively.

A comprehensive comparison of ChIP-seq experiments shows that the genomic positions occupied by TAL1 shift dramatically at progressive stages of differentiation. We compared the DNA segments occupied by TAL1 among cell types representing different stages of cell commitment and differentiation (Fig. 1A). As summarized in Table 1, TAL1 occupancy data are available for a multipotential hematopoietic precursor cell line, HPC-7 (Wilson et al. 2009), and in a population of Ter119^−^ fetal liver cells, which contain erythroid progenitors (Epro) (Kassouf et al. 2010). TAL1 ChIP-seq data were determined in our laboratory in G1E cells, G1E-ER4 + E2 (ER4) cells, Ter119^+^ erythroblasts (Ebl) from fetal liver (Wu et al. 2011), and cultured megakaryocytes from fetal liver (Pimkin et al. 2014). G1E cells were derived from mouse ES cells hemizygous for a *Gata1* knockout; these immortalized cells show many features of committed erythroid progenitor cells (Weiss et al. 1997; Welch et al. 2004; Pilon et al. 2011). A subline, G1E-ER4, was engineered to express an estradiol-dependent hybrid GATA1-ER protein, which upon hormone treatment rescues the *Gata1* deficiency and allows the cells to differentiate into erythroblasts (Weiss et al. 1997; Gregory et al. 1999). Hormonally treated G1E-ER4 cells do not complete erythroblast maturation, but preparations of primary Ter119^+^ erythroblasts contain fully differentiated erythroblasts (Fig. 1A). Cell lines were used as the source of some of the material and data in our study either because (in the case of HPC-7 cells) they provide sufficient material for ChIP, which cannot yet be obtained from primary hematopoietic precursor cells, or because (in the case of the G1E system) they allow us to study synchronized, dynamic changes dependent on a specific TF (GATA1) during erythroid maturation.

**Table 1.**
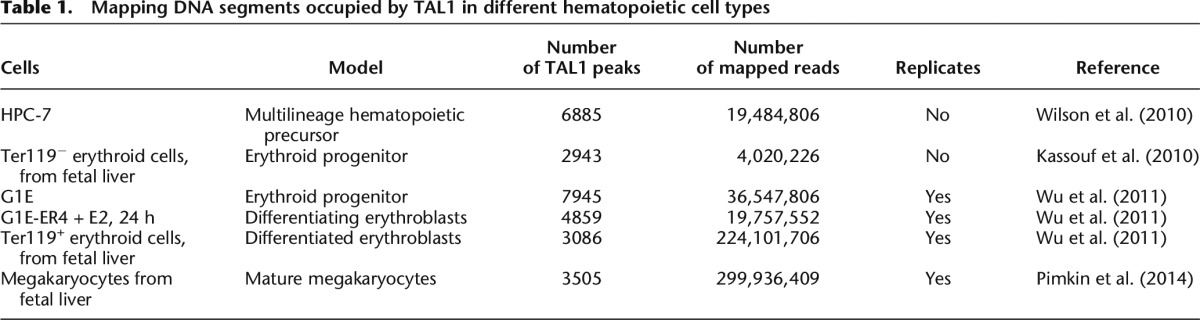
Mapping DNA segments occupied by TAL1 in different hematopoietic cell types

A comprehensive set of 18,595 TAL1-occupied DNA segments (TAL1 OSs) across myeloid hematopoiesis was constructed by taking the union of all the peak calls for these six cell types and merging overlapping segments. These were used to generate a data matrix with each TAL1 OS on a row and the value for the TAL1 ChIP-seq read count for a given cell type in each column (normalized across experiments). The sites of occupancy differ substantially, with the vast majority occurring in only one of the cell types (Fig. 1B). To ensure that our partitioning accurately reflected the signal strength at the occupied segments and was not an artifact of many segments having a signal close to a peak-calling threshold, we examined the differential occupancy in more detail in the G1E model system for erythroid differentiation. About 10,000 DNA segments were occupied by TAL1 in either the *Gata1-*null G1E progenitor cell model or in the GATA1-restored and activated ER4 cells. Of these, ∼50% were bound by TAL1 only in the absence of GATA1, ∼30% were bound before and after GATA1 was restored, and the remaining 20% were bound only after GATA1 is restored (Fig. 1C). A comparison of read counts for the TAL1 OSs in the two cell lines showed that the vast majority of peaks called only in one cell type have high tag counts in that cell line but low counts in the other, supporting the validity of the partitions (Fig. 1D). The occupancy patterns from ChIP-seq were confirmed at selected loci by ChIP-qPCR (Supplemental Fig. 1).

**Figure 2.**
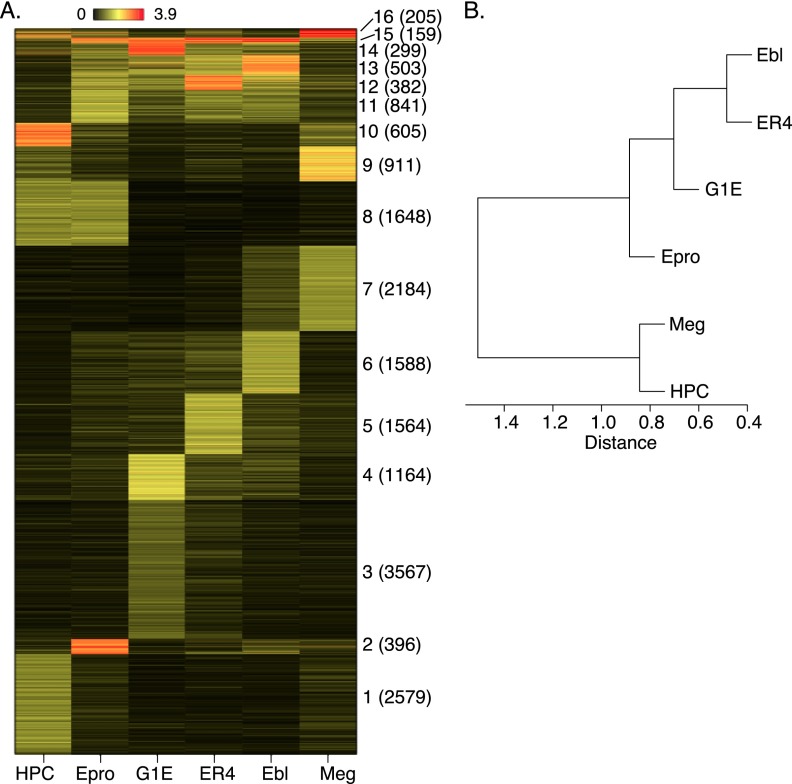
Comparison of TAL1 OSs among multiple cell types. TAL1 peaks were called individually from ChIP-seq reads in each cell type and then concatenated and merged into a union set. (*A*) The segments were clustered (*k*-means) based on the TAL1 occupancy signals in the six cell types. The clusters are dominated by binding in the following cell types: 1, HPC-7; 2, Epro (Ter119^−^ erythroid progenitors); 3 and 4, G1E; 5, ER4; 6, Ebl (Ter119^+^ erythroblasts); 7, Meg (megakaryocytes); 8, HPC-7 + Epro; 9 and 10, HPC-7 + Meg; 11, Epro + ER4 + Ebl; 12–14, all erythroid cells, Epro + G1E + ER4 + Ebl; 15, all six cell types; 16, HPC-7 + Epro + ER4 + Meg. The numbers of segments in each cluster are given in parentheses. (*B*) The correlation coefficients of the TAL1 binding signals between cell types were computed and clustered by hierarchical clustering, shown as a dendrogram.

Unsupervised clustering by *k*-means (*k* = 16) of the ChIP-seq signal strength at each TAL1 OS in the six cell types revealed the dynamics of TAL1 occupancy during differentiation (Fig. 2A). Very few TAL1 OSs were bound in all six cell types; this was estimated as 191 (1% of the total) based on the original peak calls (Fig. 1B) or as 159 using the clustering analysis (cluster 15 in Fig. 2A). Most of the TAL1 OSs in HPC-7 cells lost TAL1 during commitment to the erythroid lineage (e.g., 2579 peaks in cluster 1) (Fig. 2A). Another 1648 DNA segments bound by TAL1 in HPC-7 cells were still bound in early (Ter119^−^) erythroid progenitor cells (cluster 8) but lost TAL1 in the more differentiated erythroid cells. Conversely, most of the erythroid TAL1 OSs were not bound in HPC-7 cells (clusters 2–6 and 11–14). One group of TAL1 OSs was bound predominantly in megakaryocytes (cluster 7). Three clusters showed binding in both HPC-7 cells and megakaryocytes (9, 10, and 16), perhaps related to the “priming” of these genes in multipotential progenitors for subsequent expression in megakaryocytes (Sanjuan-Pla et al. 2013; Pimkin et al. 2014). The predominance of cell-restricted occupancy revealed by *k*-means clustering in Figure 2A was also demonstrated by hierarchical clustering and by model-based *k*-means clustering (Supplemental Fig. 2).

Even in a single committed lineage, TAL1 occupied DNA segments dynamically, according to maturational stage. Specifically, most DNA segments were bound by TAL1 predominantly in only one erythroid cell type (Fig. 2A, clusters 2–6). Even the segments bound by TAL1 in cells representing multiple stages of erythroid maturation tended to show higher binding signal in one cell type versus others (clusters 12–14). Thus most locations of TAL1 occupancy changed dramatically during commitment from HPCs to the erythroid and megakaryocytic lineages, and furthermore, many sites changed occupancy during the maturation of mono-lineage committed erythroblasts.

Pairwise correlations among the ChIP-seq signals for TAL1 OSs provide one measure of relatedness among the different cell types examined. These results, displayed as a cladogram (Fig. 2B), grouped HPC-7 cells with megakaryocytes. The cells at progressive stages of erythroid maturation formed a separate branch, with the greatest similarity between primary differentiated erythroblasts and G1E-ER4 + E2 cells, consistent with their similar profiles of gene expression (Pilon et al. 2011). While the Ter119^−^ progenitor cells fell within the erythroid clade, their TAL1 occupancy pattern was the closest of the erythroid group to HPC-7 cells. This supports the placement of the Ter119^−^ cells at an early stage of erythroid maturation (Fig. 1A) and more generally validates our experimental approach by showing that the cell types examined reflect lineage hierarchies observed during normal hematopoiesis.

### Gene targets of differential occupancy

We hypothesized that occupancy of a DNA segment by TAL1 in a particular cell type regulates the expression of one or more genes in that cell type. If so, then the genes regulated by TAL1 OSs in the different clusters should reflect lineage- or stage-specific functions. To test this hypothesis, we first partitioned TAL1 OSs into 11 groups based on how they are shared among cell types, e.g., only in HPC-7, shared between HPC-7 and Ter119^−^ erythroid progenitors, etc. (Table 2). Next we assigned genes as the likely targets regulated by each TAL1 OS. This assignment is complicated by two important factors. First, many genes are bound by TAL1 at multiple sites. While each TAL1 OS is placed into a unique category based on the pattern of occupancy in the cell types, a gene can be associated with TAL1 OSs in multiple categories. Second, determining the actual target(s) for TF-bound DNA segments is challenging because the target need not be the closest gene. Nevertheless, informative correlations between TF binding and expression have been made using simple rules for assigning targets. We used two methods. For the more inclusive method, we assigned genes as potential targets of each OS by using mouse enhancer-promoter units (EPUs), which were deduced by correlating the appearance of predicted enhancers (based on histone modification patterns) with the expression of genes (Shen et al. 2012). All genes in an EPU were assigned as potential targets of each TAL1 OS in that EPU. This approach allows genes that are within an expression-correlated genomic region to be considered as targets, but it can also assign multiple genes as targets of an individual TAL1 OS. In the second method, we assigned the gene with a transcription start site (TSS) closest to a TAL1 OS as the target. The assignment by proximity keeps a single gene as the target for each TAL1 OS but does not allow skipping of genes during assignment of targets. While both methods have limitations, we present the results that were consistent between both approaches. The genes presumptively regulated by TAL1 OSs in each cell-type partition (based on EPUs) were evaluated using the computational tool GREAT (McLean et al. 2010) for enrichment in functional categories. A selected set of 855 terms representing the common themes from this analysis, along with enrichment *Q*-values and genes for all the TAL1 OS cell-type partitions, is provided in Supplemental Table 1. The terms fell into the six major categories shown in Table 2, which also provides specific examples, *Q*-values, and presumptive target genes by major category. The results obtained when using proximity of a TSS to assign presumptive gene targets are given in Supplemental Table 2.

**Table 2.**
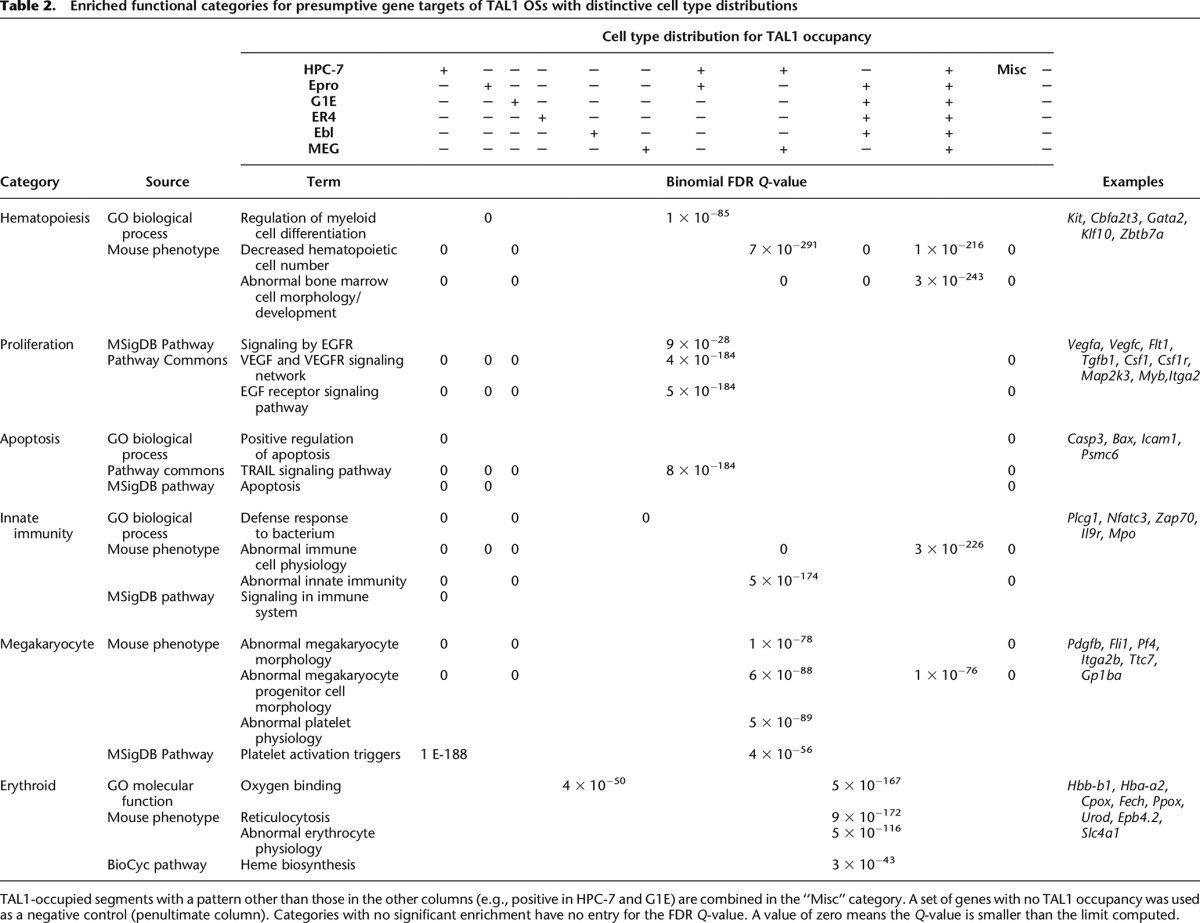
Enriched functional categories for presumptive gene targets of TAL1 OSs with distinctive cell type distributions

The presumptive gene targets of HPC-7-specific TAL1 occupancy were highly enriched for functions associated with hematopoiesis, proliferation, and apoptosis. Examples of hematopoietic genes (Table 2) are *Kit*, encoding the receptor for stem cell factor, and *Cbfa2t3*, encoding a core-binding factor whose ortholog in humans is rearranged in some leukemias. Examples of presumptive target genes associated with proliferation include those encoding growth factors and receptors such as VEGFA and its receptor FLT1, TGFB1 and TGFBR1, and CSF1 and CSF1R. Genes encoding proteins in signaling pathways for proliferation, such as MAP2K3 and MYB, also are preferentially bound by TAL1 in HPC-7 cells. Terms associated with apoptosis were also enriched in these presumptive target genes. Several examples of lineage-specific occupancy of genes in these categories are shown in Supplemental Figure 3 (*Vegfa*, *Vegfc*, *Kit*, *Myb*).

Binding of TAL1 in HPC-7 cells and other less differentiated cells could participate in lineage priming, i.e., the expression of lineage-specific genes in multilineage progenitors (Mansson et al. 2007; Pina et al. 2012). Genes that are presumptive targets of TAL1 occupancy in HPC-7 cells, as well as in Ter119^−^ progenitors, were highly enriched for functions associated with the differentiated myeloid cells, perhaps reflecting the maintenance of multiple lineage potentials. The HPC-7 cells can be induced to differentiate into several myeloid cell types such as granulocytes and monocytes (Pinto do O et al. 1998), and terms associated with innate immunity are strongly enriched for the presumptive targets of TAL1 in these cells (Table 2). This could indicate that HPC-7 cells, and by inference multilineage hematopoietic progenitor cells, maintain expression of some genes characteristic of the differentiated progeny cells through the binding of TFs such as TAL1.

**Figure 3.**
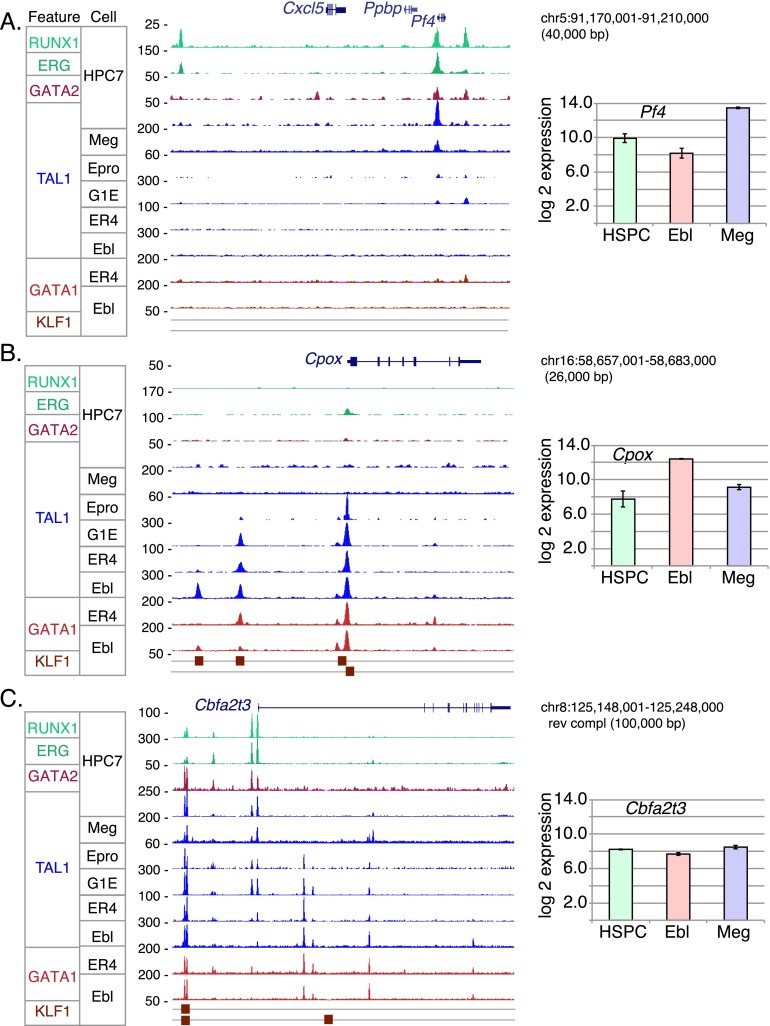
Loci illustrating shifts in patterns of TAL1 occupancy and co-occupancy by other transcription factors among the six differentiation stages. Each panel shows on the *left* the signal track for each indicated transcription factor binding in the designated cell type. Signal for TAL1 binding is blue; for GATA factors, red; and those for other factors, green. Peak calls for KLF1 in erythroblasts are shown from two different sources; the *upper* boxes are from Tallack et al. (2010), and the *lower* boxes are from Pilon et al. (2011). Direction of transcription for each gene is *left* to *right*. The graphs on the *right* show levels of expression in hematopoietic stem progenitor cells (HSPCs), which are a proxy for expression in HPC-7 cells, erythroblasts, and megakaryocytes (Pimkin et al. 2014). (*A*) The *Pf4* gene (encoding platelet factor 4) illustrates TAL1 binding in megakaryocytes and in HPC-7, not in erythroid cells. (*B*) The *Cpox* gene (encoding coproporphyrinogen oxidase) is bound almost exclusively in erythroid cells. (*C*) The *Cbfa2t3* gene is bound at multiple locations, which show a diversity of patterns across differentiation.

The case for lineage priming by TAL1 is quite strong for megakaryocytic genes. Several pathways, phenotypes, and Gene Ontology (GO) terms associated with megakaryocytes were enriched for the sets of genes that are presumptive targets of TAL1 OSs observed in both HPC-7 cells and in megakaryocytes (Table 2). Thus, these genes are bound not only in the mature, differentiated cells where the gene product participates in platelet specific functions but also in the multilineage progenitor cells. This precocious binding in progenitor cells could be mediating early expression of these genes, which are subsequently induced to higher levels in megakaryocytes. An example is the gene *Pf4*, encoding the precursor to platelet factor 4, and the adjacent gene *Ppbp*, encoding pro-platelet basic protein. Using gene expression data from a population of hematopoietic precursors that is not lineage committed (Sca1^+^ Lin^−^), for which HPC-7 cells serve as a proxy, and from primary erythroblasts and cultured megakaryocytes (Pimkin et al. 2014), we found that *Pf4* (Fig. 3A) and *Ppbp* (data not shown) were expressed in multilineage precursor cells and were further induced in megakaryocytes, but expression levels were lowered in erythroblasts. Similarly, TAL1 was bound near the TSS of *Pf4* in HPC-7 cells and megakaryocytes. A weak signal for TAL1 was seen in erythroid progenitors, and this declined further as erythroid maturation progressed (Fig. 3A). Moreover, in HPC-7 cells, this DNA segment was bound by additional TFs (GATA2, ERG, and RUNX1) that are part of the heptad containing TAL1 (Wilson et al. 2010); some of these components were also bound to distal sites. This pattern of TAL1 occupancy is consistent with its role in lineage priming of megakaryocyte-specific genes in multilineage progenitors. Numerous other megakaryocyte genes, such as *Gp1ba* and *Pdgfb*, appear to be primed in HPC-7 cells via TAL1 occupancy (Supplemental Fig. 4).

In contrast, the presumptive target genes of the TAL1 OSs that are shared among Epro, G1E cells, ER4 cells, and Ebl were significantly associated with functions and phenotypes that are characteristic of the erythroid lineage, such as heme biosynthesis, reticulocytosis, and erythrocyte physiology (Table 2). This association indicates that the set of TAL1 OSs that is stably occupied through erythroid maturation is used to maintain the expression of erythroid lineage-specific genes. The fact that these sites are not typically bound in HPC-7 cells indicates that lineage priming is not as prevalent for erythroid genes as it is for megakaryocytic ones. A striking example of TAL1 occupancy restricted to the erythroid lineage but present at multiple stages of maturation is the flanking region of *Cpox*, encoding the heme biosynthetic enzyme coproporphyrinogen oxidase. TAL1 binding close to the TSS was observed in committed erythroid progenitors but not in HPC-7 cells or megakaryocytes. Additional sites were bound in cells representing progressively more mature erythroblasts, where *Cpox* was highly expressed (Fig. 3B). GATA1 and KLF1 were also bound to the TAL1 OSs in erythroblasts. These additional sites of binding by TAL1 and other hematopoietic TFs may serve to maintain *Cpox* expression as most genes become repressed during the later stages of erythroid maturation. A similar erythroid-specific pattern of occupancy is seen for many erythroid genes (Table 2); additional examples of *Fech* and *Hbb-b1* are shown in Supplemental Figure 5.

Often, multiple DNA segments of the same gene were bound by TAL1 and associated proteins with distinctive patterns of protein occupancy. Consider the gene *Cbfa2t3*, encoding the protein CBFA2T3 (also known as ETO2, MTG16, and MTGR2), a co-repressor (Hug and Lazar 2004) implicated in hematopoietic regulation (Schuh et al. 2005). *Cbfa2t3* was bound by TAL1 at a minimum of 11 sites. Some of these OSs were bound in all cell types examined, while others increased or decreased progressively during erythroid maturation (Fig. 3C). This diversity of binding patterns indicates a complex set of regulatory regions that are utilized dynamically during hematopoiesis. Remarkably, these dynamic changes in occupancy are not accompanied by large changes in expression of *Cbfa2t3*, suggesting that distinct sets of TF-bound DNA segments can be utilized in different lineages to achieve similar levels of expression. Similar complex patterns of occupancy were observed for multiple genes, including those encoding TFs such as RUNX1T1 (CBFA2T1, ETO), GATA2, and RUNX1 (Supplemental Fig. 6). The binding of TAL1 to distinctive sites in different cells for a given gene means that, in our analysis, the gene was placed in multiple categories of presumptive targets for TAL1, which contributes to the appearance of functional enrichment terms in unexpected cell types, such as enrichment for megakaryocytic functions and innate immunity in G1E cells (Table 2). TAL1 binding in erythroid cells to genes expressed in other lineages suggests that TAL1 may be playing a repressive role in these cases. Indeed, the genes contributing to the enrichment for megakaryocytic function in targets of TAL1 occupancy in erythroid cells tended to be repressed in erythroblasts (Supplemental Fig. 7). Thus, despite the confounding effects of multiple TAL1 OSs per gene and multiple targets for some OSs, the analysis by GREAT produced several meaningful categories of functional enrichments for cell-specific occupancy.

### Determinants of differential occupancy: histone modifications

**Figure 4.**
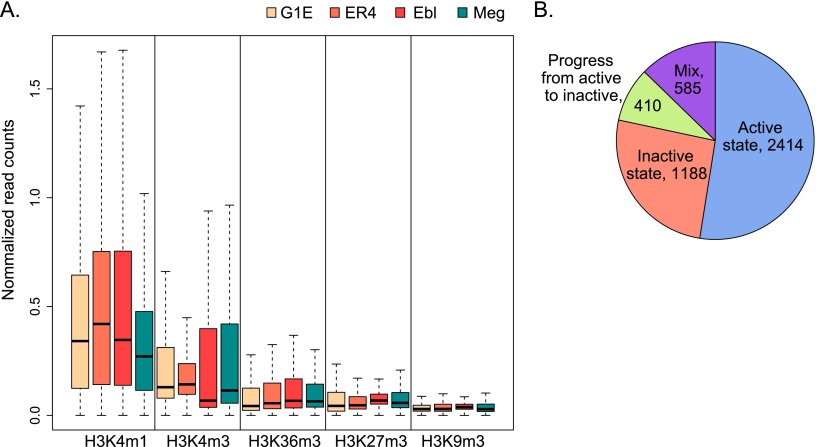
Histone modifications and chromatin states on TAL1 OSs. (*A*) Levels of five histone modification ChIP-seq signals (shown by box plots) in G1E, G1E-ER4 + E2, Ter119^+^ erythroblasts, and megakaryocytes were computed on the DNA segments occupied by TAL1 OSs in each cell type. (*B*) Chromatin states were determined using chromHMM (Ernst and Kellis 2010; Ernst et al. 2011) after learning the model from the five histone modifications in multiple cell types (Cheng et al. 2014). The pie chart shows the number of HPC-7-only TAL1 OSs that carry the designated pattern of chromatin states in the three erythroid cell types.

The DNA segments bound by TAL1 reside in chromatin with histone modifications associated with gene activation, such as H3K4 mono- and trimethylation and H3K36 trimethylation, with little or no signal for the repressive modifications H3K27 trimethylation or H3K9 trimethylation (Fig. 4). This analysis was conducted for TAL1 OSs in G1E cells, ER4 cells, Ter119^+^ erythroblasts, and megakaryocytes, for which the histone modification data are available.

While no histone modification data are available for the HPC-7 cells or Ter119^−^ erythroid progenitors, we were able to assess whether the DNA segments bound by TAL1 only in HPC-7 cells change to an inactive chromatin state in erythroid cells. The genomes of G1E, ER4, and Ter119^+^ cells were segmented based on combinations of histone modification signals using a nine-state model generated by chromHMM (Ernst and Kellis 2010; Ernst et al. 2011; Wu et al. 2011; The Mouse ENCODE Consortium et al. 2014). Each of the 4597 peaks called as bound by TAL1 only in HPC-7 cells was then assigned to one of the nine chromatin states in the three erythroid cell types. To simplify the 27 possible combinations of the nine chromatin states in three cell types, each TAL1 OS was then placed in a summary category of active chromatin—defined as being in a state enriched for H3K4me1, H3K4me3, or H3K36me3—or inactive chromatin—defined as being in a state enriched for H3K27me3, H3K9me3, or no histone modification. Of the DNA segments bound by TAL1 only HPC-7 cells, we found that about half remained in an active chromatin state in the erythroid cell types (Fig. 4B). About 28% of them had the opposite fate, being found in inactive chromatin in all three erythroid cell types. Another 9% progressed to inactive states across the series G1E > G1E-ER4 + E2 > Ter119^+^ cells. The remaining 14% fell into a mix of active or inactive states in the three cell types. Making the likely assumption that the DNA segments were in active chromatin states when bound in HPC-7 cells, these results indicate that almost 40% of the DNA segments that lose occupancy by TAL1 are associated with inactivation of the chromatin.

### Determinants of differential occupancy: binding site motifs and binding by other proteins

Given that active chromatin states are preferred for TF binding in all cell types, we searched for signals that could help determine differential occupancy by TAL1 across cell types. We hypothesized that the relocation of TAL1 occupancy during differentiation could be driven by other TFs bound to their cognate motifs within the TAL1 OSs, and we tested it by searching for matches to binding site motifs that are distinctive for different cell types.

**Figure 5.**
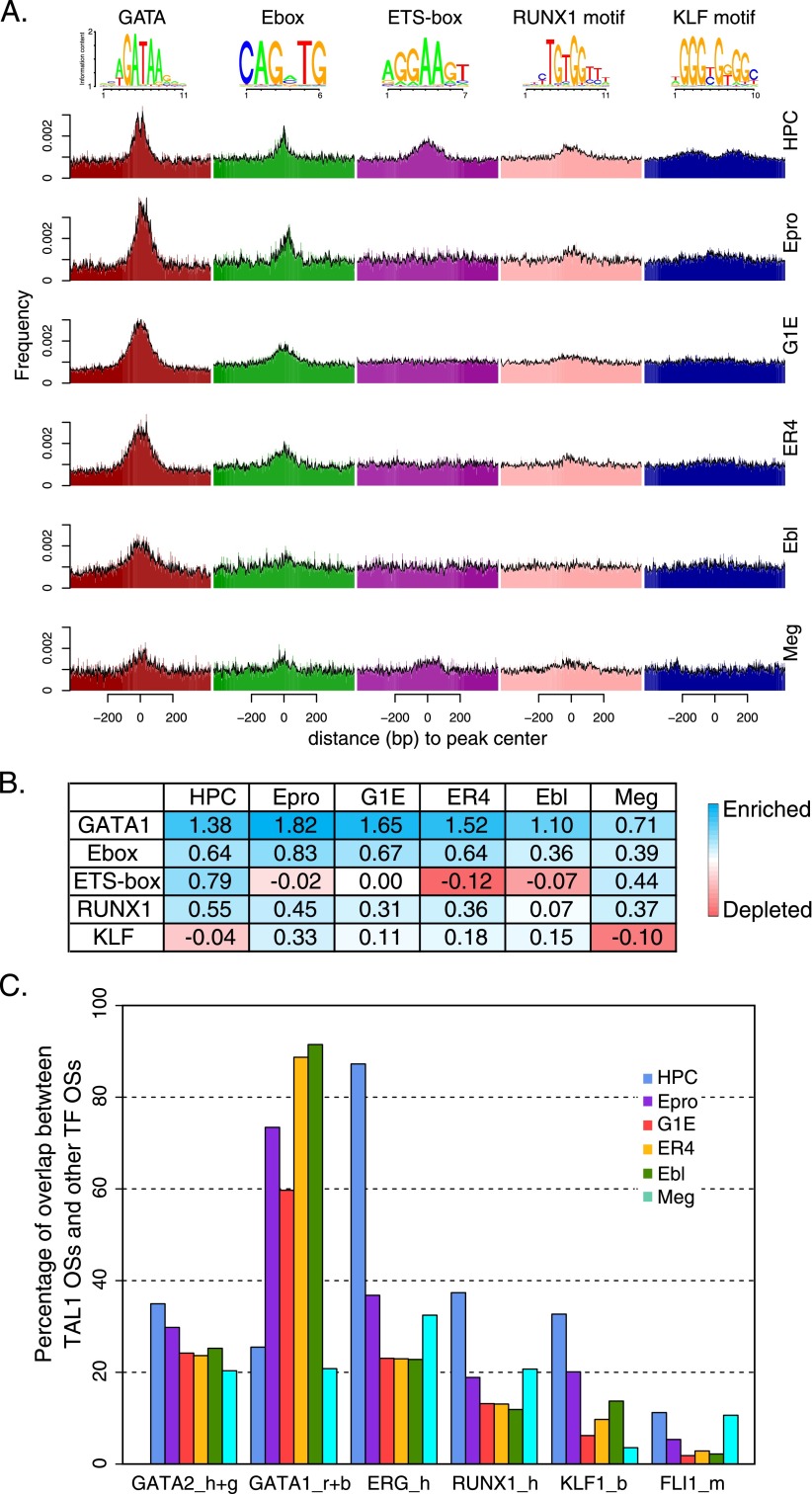
Comparison of enrichment of transcription factor binding site motifs and co-occupancy on TAL1 OSs in six hematopoietic cell types. (*A*) The locations of five motifs (logos at the *top* of the panel) on 1-kb intervals (only 800 bp is displayed here) centered on TAL1 OS peak centers were found by FIMO in each of the six cell types. The distribution of the locations is plotted by both histograms (colored bars) and density plots (black curves on *top* of histograms). (*B*) Enrichment or depletion of each motif in the TAL1 OSs for each cell type. (*C*) Fraction of TAL1 OSs (cell types indicated by color key) bound by the transcription factor indicated on the *x*-axis. Cell types for the transcription factor binding are abbreviated: h, HPC-7; g, G1E; r, G1E-ER4 + E2; b, erythroblast; m, megakaryocytes.

To find the enriched motifs and their distribution patterns on the different sets of TAL1 binding sites, we first used MEME-ChIP and related tools (Machanick and Bailey 2011) to generate a list of known TF binding site motifs enriched in the occupied segments in each cell type (Supplemental Table 3). Five motifs predominated in the MEME results, corresponding to binding site motifs for GATA factors, bHLH proteins (E-box), ETS proteins (ETS-box), RUNX proteins, and Krüppel-like factors (KLFs) (Fig. 5). We then used FIMO (Grant et al. 2011) to locate all instances of these motifs in each 1-kb interval centered on a TAL1 OS mid-point. The distribution of motif instances was summarized by a histogram (Fig. 5A), and more detailed views of the patterns of motif instances across the TAL1 OSs were generated as dot plots (Supplemental Fig. 8; Ozdemir et al. 2011). The motif enrichment score was computed as the logarithm (base 2) of the odds ratio determined by the number of motif instances in the central 100 bp compared to the number in the rest of the OS, in each case normalized for the frequency of motif instances in comparably sized random DNA segments (Fig. 5B).

The preferred DNA binding site for a heterodimer of TAL1 with E-proteins in vitro is CAGVTG. A position-specific weight matrix (PWM) derived from the MEME analysis matched this consensus, and we used it to find instances of this particular E-box in the TAL1 OSs. This motif was enriched in binding sites from all six cell types (Fig. 5B), showing a peak in occurrence at the center of the occupied DNA segments (Fig. 5A; Supplemental Fig. 8), as expected for a determinant of occupancy.

Surprisingly, the level of enrichment for the TAL1 E-box motif was only about half that observed for the motif corresponding to a GATA factor binding site, which was the most strongly enriched of any motif (Fig. 5A,B). This motif showed a substantial preference for the centers of the TAL1 OSs (Fig. 5A; Supplemental Fig. 8). This pattern was observed for the TAL1-bound DNA segments in all six cell types, suggesting a consistent and frequent co-occupancy of TAL1 and GATA factors. However, the same GATA factor cannot account for these enrichments, since hematopoietic precursor and erythroid progenitor cells express GATA2, which is replaced by GATA1 during erythroid differentiation. Hence we interpret the predominance of the GATA binding site motif in the TAL1 OSs as reflecting the binding of TAL1 with GATA2 in earlier stages (represented by HPC-7, Epro, and G1E cells) and GATA1 in more differentiated stages (represented by ER4 and Ebl).

The interpretation of the motif enrichment as reflecting co-occupancy was tested by looking for overlap between the TAL1 OSs in each cell type and occupancy data for other TFs. This analysis strongly supported a role for GATA2 and GATA1 in determining TAL1 occupancy. As previously reported (Wilson et al. 2010), ∼30% of TAL1 OSs in HPC-7 cells were also bound by GATA2, and we found that this fraction of overlap with DNA segments occupied by GATA2 (in HPC-7 cells or G1E cells) continued with only a small decline across the erythroid and megakaryocytic cell types (Fig. 5C). The overlap with GATA2 OSs persisted even for TAL1 occupancy in ER4 cells and erythroblasts, in which GATA2 is no longer present, suggesting that binding by GATA2 in early progenitor cells marked some of the sites that would later be occupied by TAL1 in mature cells. Co-occupancy with TAL1 in erythroid cells was even higher for GATA1, with overlaps ranging from 60% to almost 90% (Fig. 5C). Specific examples are shown for *Pf4*, *Cpox*, and *Cbfa2t3* (Fig. 3) and for other genes (Supplemental Figs. 4–6). The “co-occupancy” between GATA1 and TAL1 was observed even in some cell types that have no (G1E) or low (HPC, Epro) GATA1. In these cases, TAL1 was already bound to a site to which GATA1 would normally bind later. Presumably these sites were marked by other proteins, such as GATA2 and appropriately modified histones, to favor binding by TAL1. Thus binding by GATA factors is likely to be a strong determinant of TAL1 occupancy in all the cell types examined. Indeed, from the levels of enrichment of motifs, it appears to be a stronger determinant than the TAL1 E-box.

The distributions of three other motifs were distinctive among the cell types, providing candidates for proteins that may aid in discriminating sites of occupancy by TAL1. The ETS-box, which is recognized by ETS proteins such as ERG, FLI1, and SPI1 (also known as PU.1), was enriched only in TAL1 OSs from HPC-7 cells and megakaryocytes, and it was depleted in TAL1 OSs from erythroblasts (Fig. 5A,B; Supplemental Fig. 8). This pattern suggested that co-occupancy by ETS proteins favors TAL1 occupancy in multilineage hematopoietic progenitors. We tested this hypothesis by examining the overlap between the TAL1 OSs in each of the six cell types and the DNA segments bound by ERG in HPC-7 cells. Not only did this confirm the substantial cobinding of TAL1 and ERG in HPC-7 cells (Wilson et al. 2010), but we also found that the fraction of overlap decreased dramatically for TAL1 OSs in the more mature erythroid cells (Fig. 5C). Furthermore, the overlap with FLI1-bound sites implicate this ETS protein in helping to determine occupancy by TAL1 in megakaryocytes (Fig. 5C; see also Pimkin et al. 2014). Another discriminatory motif, the binding site for RUNX1, was enriched in TAL1 OSs from HPC-7 cells and to a lesser extent in TAL1 OSs from Epro cells and megakaryocytes (Fig. 5B), and the overlap with RUNX1 binding (in HPC-7 cells) was higher for TAL1 OSs in these cells than in more mature erythroblasts (Fig. 5C). Thus both the motif enrichment and overlap in TF binding strongly support binding by ETS proteins and RUNX1 as positive determinants of TAL1 occupancy in multilineage progenitor cells, early erythroblasts and megakaryocytes.

The KLF binding site motif was also enriched in TAL1 OSs in HPC-7 cells, but it tended to localize away from the center of the TAL1-bound DNA segment (Fig. 5A). The measure of enrichment we adopted emphasized motifs at the center of the bound segment, and thus this measure showed a moderate depletion in HPC-7 cells (Fig. 5B), whereas over the entire bound segment the KLF motif is enriched. The KLF motif also was moderately enriched in TAL1 OSs from erythroid cells. The patterns of KLF motifs in the TAL1 OSs leads to the surprising inference that binding by a member of the KLF family has a positive effect on TAL1 binding in HPC-7 cells. KLF1 occupancy has been studied genome-wide in erythroblasts (Tallack et al. 2010; Pilon et al. 2011), where this protein is present at high levels. We utilized these data to test the possible involvement of KLF proteins in TAL1 binding, assuming that some sites bound by KLF1 in erythroid cells were previously bound by a paralogous member of the KLF family in progenitor cells. This assumption is analogous to the overlap seen in GATA1 (erythroid cells) and GATA2 (earlier progenitor cells) binding (Anguita et al. 2004; Bresnick et al. 2010; Wu et al. 2011; Suzuki et al. 2013). As predicted from the motif enrichment, KLF binding (Pilon et al. 2011) overlapped TAL1 occupancy in HPC-7 cells (Fig. 5C). Thus both lines of evidence implicate binding by a KLF family member as a contributor to TAL1 occupancy in multilineage progenitors cells.

### Changes in position of TAL1 occupancy during GATA1-induced erythroid differentiation

**Figure 6.**
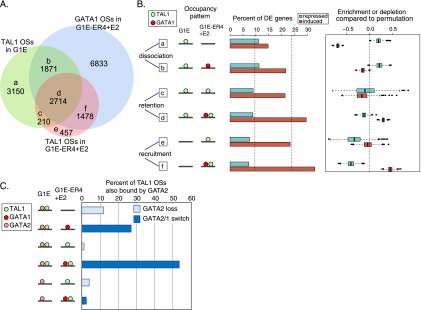
Changes of TAL1 occupancy in GATA1-induced erythroid differentiation. (*A*) Venn diagram showing TF-occupied DNA segments partitioned by occupancy by TAL1 in G1E and ER4 cells and by GATA1 in ER4 cells. (*B*) Expression response of presumptive target genes. Genes were associated with TAL1 OSs based on their colocalization in EPUs. For each of the six TAL1 occupancy patterns, the percentages of the differentially expressed (DE) presumptive targets that are induced or repressed are shown as bar plots. The average percentages obtained after 1000 shufflings of the TAL1 OS category (groups a–f) within the matrix are shown by the dotted lines in the bar plot (*left* for repressed, *right* for induced). For each of the 1000 permutations, the true percentage of DE genes was divided by the percentage from the shuffled data set. The distributions of the log_2_ (true percentage/shuffled percentage), shown as box plots, give an estimate of the relative enrichment or depletion for induction or repression of the presumptive gene targets in each TAL1 OS category. (*C*) Role of GATA2 in TAL1 occupancy. For the subset of TAL1 OSs that is also bound by GATA2 in G1E cells, the percentages that fall into each of the TAL1 OS partitions defined in panel *A* are plotted. The DNA segments bound by both TAL1 and GATA2 in groups a, c, and e in panel *A* are GATA2 loss sites, whereas those in groups b, d, and f are GATA switch sites.

Given the strong tendency for TAL1 and GATA factors to bind the same DNA segments, we used the *Gata1* gene complementation system (G1E and ER4 cell lines) to examine directly the effects of GATA1 restoration on TAL1 binding. Most TAL1 OSs in either G1E or ER4 cell lines (6063, or 60%) overlapped with the DNA segments bound by GATA1 in ER4 cells (Fig. 6A). The connection between GATA1 and TAL1 was also revealed by the strong correlation in signal strength for TAL1 and GATA1 ChIP-seq results in ER4 cells (Supplemental Fig. 9C), confirming on a genome-wide scale the results seen with occupancy on mouse chromosome 7 (Cheng et al. 2009). The seven occupancy categories defined by overlaps of peak calls (Fig. 6A) were strongly supported by the distributions of binding signal strengths for TAL1 OSs in each partition (Supplemental Fig. 9B).

In the G1E cell *Gata1* knockout and rescue system, we can distinguish three types of response by TAL1 to restoration of GATA1: dissociation, retention, or recruitment of TAL1. Furthermore, each response can be a direct effect of GATA1 binding or indirect (Fig. 6B). Thus, the TAL1 OSs were partitioned initially by response category and then further by whether this DNA segment was also occupied by GATA1 in ER4 cells, indicating a direct effect on TAL1, or not, indicating an indirect effect (groups a–f illustrated in Fig. 6B). The dynamic occupancy patterns for TAL1 and GATA1 were strongly associated with the expression response of presumptive target genes. We reexamined this issue using the RNA-seq data for G1E and G1E-ER4 + E2 cells (Wu et al. 2011; Paralkar et al. 2014) and employing EPUs for assigning presumptive gene targets. For each TAL1 OS, all genes with a TSS within the same EPU were considered as potential targets, and the fraction of those genes that were induced or repressed was computed. The data for all the TAL1 OSs in each response category were combined to generate an average (shown as the percent of differentially expressed [DE] genes), which allows a comparison of whether the presumptive targets tend to be induced or repressed (bar plot in Fig. 6B). To assess whether these averages represent enrichment for the response relative to that of all the presumptive TAL1 targets, a shuffling strategy was employed to generate the distribution of enrichment (or depletion) values compared with permutations (boxplots in Fig. 6B).

The genes that are presumptive targets of the 4192 DNA segments co-occupied by GATA1 and TAL1 in ER4 cells were strongly associated with induction, regardless of the presence or absence of TAL1 on the DNA segment in G1E cells. Group d contains the 2714 instances of TAL1 retention, whereas group f contains the 1478 cases of direct recruitment in response to GATA1 restoration. Both groups show more frequent induction than repression, and these results represent enrichment for induction and depletion for repression (Fig. 6B). In contrast, the presumptive gene targets of the 5021 DNA segments at which GATA1 led to dissociation of TAL1 (i.e., bound by TAL1 in G1E but not ER4 cells) were enriched for repression and depleted for induction. This was the case whether the dissociation of TAL1 was inferred to be a direct (1871 TAL1 OSs in group b) or indirect (3150 TAL1 OSs in group a) effect (Fig. 6B). Two additional occupancy categories were composed of DNA segments at which TAL1 was either retained (210 TAL1 OSs in group c) or recruited (457 TAL1 OSs in group e) after GATA1 restoration, but were inferred to be indirect effects because GATA1 was absent at those DNA segments. The presumptive gene targets of these two categories of TAL1 OSs were depleted for both induction and repression. These results strongly confirm and extend the positive association of induction with co-occupancy by GATA1 and TAL1 (Cheng et al. 2009; Tripic et al. 2009; Soler et al. 2010). Furthermore, they show that loss of TAL1 occupancy is negatively associated with induction but positively associated with repression.

**Figure 7.**
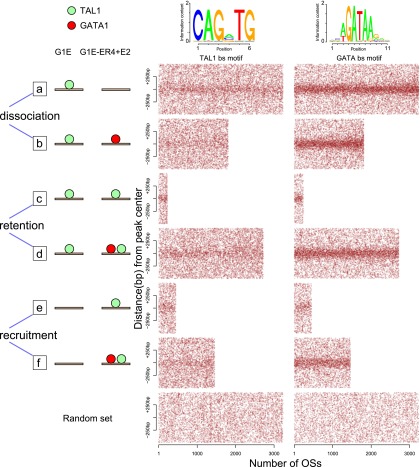
Distribution of GATA and TAL1 binding site motif instances in different categories of TAL1OSs. The location of DNA sequences matching the binding site motif for GATA factors or TAL1 within 500 bp from the center of each OS is indicated by a red dot. TAL1 OSs were separated into six categories based on the cell types in which they are bound and their co-occupancy by GATA1, shown by diagrams on the *left*, with green and red disks representing TAL1 and GATA1 occupancy, respectively. In each panel, the OSs were sorted from *left* to *right* by increasing occupancy level of TAL1 in the corresponding cell line.

Surprisingly, the sharp differences in dynamics of occupancy by TAL1 and GATA1 in these six groups of TAL1 OSs were actuated on rather similar distributions of TF binding site motifs. We employed dot-plots to show the positions of all motif instances in each TAL1 OS (1-kb intervals centered on the peak mid-point). Motifs that contribute strongly to occupancy are expected to generate dense concentrations of motif instances close to peak centers and to do so throughout most of a data set (Ozdemir et al. 2011). Consistent with recent reports (Kassouf et al. 2010) and the strong enrichment of GATA motifs seen for the entire set of TAL1 OSs (Fig. 5), we found that the TAL1 OSs that were also bound by GATA1 had a strong enrichment for the GATA binding site motif around the peak centers (Fig. 7, groups b, d, f). However, the TAL1 OSs that were not co-occupied by GATA1 also had a strong enrichment for the GATA binding site motif (Fig. 7, groups a, c, e). The canonical TAL1 binding site motif was also enriched toward the peak centers of TAL1 OSs in each category, albeit considerably less strongly than the GATA binding site motif.

The presence of GATA binding site motifs in the centers of TAL1 OSs that were not bound by GATA1 suggests that GATA2 could also direct TAL1 binding in G1E cells. GATA2 bound at specific sites in erythroid progenitor cells can be replaced by GATA1 in maturing erythroblasts; these GATA switch sites have been implicated in repression during maturation (Jing et al. 2008; Bresnick et al. 2010; Dore et al. 2012; Suzuki et al. 2013). To examine the role of GATA2 and GATA switch sites in TAL1 occupancy, we examined the ∼4000 DNA segments bound by GATA2 in G1E cells (a lower-bound estimate) (Wu et al. 2011) and found that 1140 TAL1 OSs overlapped with the GATA2 OSs. These comprised from 2% to 22% of the TAL1 OSs in the categories defined in Figure 6A (Supplemental Fig. 10). Of these TAL1 OSs that were also bound by GATA2, most were at GATA switch sites that retained TAL1 after the switch (Fig. 6C). These were part of group d, which was enriched for induction of the presumptive target genes (Fig. 6B). Another 27% were at switch sites that lost TAL1 after GATA2 was replaced by GATA1, which is a mechanism for repression (Fig. 6B, group b; Jing et al. 2008; Bresnick et al. 2010). Some of the GATA2 OSs ascertained in G1E cells were not bound by GATA1 in ER4 cells; we refer to these as GATA2 loss sites. These were most frequent in group a, in which TAL1 dissociated upon GATA1 restoration (Fig. 6B,C). Again, the presumptive targets for this group were enriched for repression.

## Discussion

**Figure 8.**
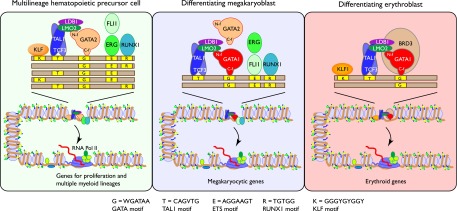
Model for differential occupancy by TAL1 in multilineage versus erythroid cells. The groups of proteins that co-occupy DNA in the two cell types are shown, along with cognate binding site motifs (yellow boxes, identity of each motif is listed at the *bottom*) along the DNA (long brown rectangle). The different arrangements of motifs signify the diversity of motifs seen in TAL1-occupied segments and emphasize the predominance of the GATA motif. The proteins TAL1:TCF3, LMO2, LDB1, and GATA1 form a multiprotein complex (Rodriguez et al. 2005); the other proteins shown are in proximity when bound to DNA. Additional proteins recognizing the same motif as other members of the TF family and bound at some sites (e.g., GATA2 and ERG in megakaryocytes) are also shown. Specific binding by TFs occurs within accessible DNA in chromatin that itself has histone modifications associated with gene activity (green and yellow circles representing methylation of lysines on H3 and acetylation of H4). The complex of proteins including TAL1 can exert positive effects on expression (curved arrow as shown) on recruitment and release of RNA polymerase for active transcription on induced genes. Other TAL1-containing complexes can exert negative effects (not shown). Cell-specific binding of TAL1 and associated proteins target different cohorts of genes.

TAL1 is a major regulator at multiple stages of hematopoiesis. Our comparative study of TAL1 occupancy shows that it contributes to the regulatory regimen in markedly different cell types by binding to different genomic DNA sites at progressive stages of hematopoietic differentiation into specific lineages and their subsequent maturation. We analyzed globally the binding of TAL1 and expression profiles in six mouse cell types representing distinct stages of hematopoiesis. The sites of occupancy changed dramatically during the shift from HPC-7 cells, a model for a multilineage hematopoietic precursor cell, to cells committed to either the erythroid or megakaryocytic lineage. These changes in sites occupied by TAL1 were linked to alterations in the gene expression profiles of the different cell types. Genes that are likely targets for regulation by TAL1 in HPC-7 cells were enriched in functions associated not only with hematopoiesis but also with the specific differentiated progeny derived from these multilineage precursors. Thus, a subset of the sites occupied by TAL1 in multilineage progenitors remains bound in megakaryocytes, and the presumptive target genes are expressed at high levels in megakaryocytes. This represents a clear example of lineage priming that confirms other recent work (Sanjuan-Pla et al. 2013; Pimkin et al. 2014). In contrast, genes that are likely targets for regulation by TAL1 in cells committed to the erythroid lineage are enriched for erythroid-specific functions. Furthermore, the sites of occupancy continue to change during erythroblast maturation, leading to induction of erythroid genes and repression of genes expressed in alternate lineages and progenitors. These data support a model in which TAL1 regulates distinct cohorts of genes in different cell types by binding to different *cis*-regulatory modules (Fig. 8).

Features that determine cell-specific binding by TAL1 are becoming defined, but we are still far from understanding them completely. TAL1 binds to DNA segments that are in permissive chromatin, i.e., having histone modifications associated with active expression or regulation (Fig. 8), as has been seen for GATA1 (Zhang et al. 2009) and other TFs (Arvey et al. 2012; Kundaje et al. 2012). We have proposed that the permissive chromatin states are established no later than lineage commitment (Wu et al. 2011), based on the very limited changes observed in chromatin states during the substantial changes in gene expression during maturation after erythroid commitment (Wong et al. 2011; Wu et al. 2011). In our current study, we examined earlier stages of differentiation to infer some changes in chromatin states during lineage commitment. By assuming that TAL1 binding in multipotential HPC-7 cells is in permissive chromatin (as it is in the daughter lineages), we deduced that almost 40% of the sites bound by TAL1 only in HPC-7 cells (i.e., no longer bound in erythroid cells) shift to inactive chromatin in erythroid cells. Thus shifting to a nonpermissive chromatin state accompanies some of the changes in TAL1 occupancy that occur during commitment to the erythroid lineage. Another half of the DNA segments bound by TAL1 only in HPC-7 cells show the opposite trend. They stay in active chromatin in erythroid cells, which may reflect continued occupancy by other factors or a cellular memory of occupancy.

TAL1 binds to appropriate sites within permissive chromatin largely by its interactions with other TFs such as GATA factors. While a subset of E-boxes (CAGVTG) contributes to the specific binding by TAL1 (as a heterodimer with TCF3), we find that the GATA binding site motif is a much stronger determinant of specific occupancy. Previous studies revealed a role for the GATA motif and GATA factors in binding of TAL1 to some sites (Ono et al. 1998; Cheng et al. 2009; Tripic et al. 2009; Kassouf et al. 2010), and our current results emphasized that this motif is the dominant one in almost all categories of TAL1 binding. Genetic rescue experiments showed that the DNA-binding domain was not required for many TAL1 functions (Porcher et al. 1999). Recent structural analysis demonstrated that the bHLH domains of TAL1:TCF3 do not make extensive contacts with the E-box in DNA, and the interactions with GATA factors, via LMO2, were inferred to be major determinants of specific occupancy (El Omari et al. 2013). These multiple lines of evidence show that TAL1 is directed to most specific binding sites not through a high affinity interaction with an E-box, but rather by interactions with other proteins. GATA2 and GATA1 are implicated at many (up to 90%) of the TAL1-bound sites in normal hematopoietic cells, and GATA3 (Ono et al. 1998) along with ETS1 and RUNX1 (Palii et al. 2011) can direct TAL1 binding when it is aberrantly expressed in T-cell leukemias. Presumably other TFs facilitate TAL1 binding in various other cell types, such as neurons (Achim et al. 2013) and endothelial cells (Visvader et al. 1998), where it exerts essential functions. While the DNA binding domain of TAL1 is not needed for its function at some bound sites, at others the DNA binding domain does interact with the E-box (El Omari et al. 2013). This latter subset of sites could contribute to the observed enrichment for the canonical TAL1 binding site motif; these may have functions distinct from those TAL1 OSs without the canonical motif.

The cobinding of TAL1:TCF3 and GATA factors can occur by the binding of large multiprotein complexes. A pentameric complex composed of the TAL1:TCF3 heterodimer and GATA1 (or GATA2) connected by the bridge proteins LMO2 and LDB1 (Wadman et al. 1997) is present in erythroid cells (Rodriguez et al. 2005). When bound to DNA, this complex can account for much of the TAL1-GATA factor co-occupancy observed in all the cell types examined, i.e., HPC-7 cells (Wilson et al. 2010), megakaryoblasts (Tijssen et al. 2011; Dore et al. 2012; Pimkin et al. 2014), and erythroblasts (Fig. 8; Cheng et al. 2009; Soler et al. 2010; Wu et al. 2011).

The differential occupancy at progressive stages of differentiation and in distinct lineages is further directed by the binding of additional, lineage-specific TFs. We confirmed that TAL1-bound sites in HPC-7 cells were frequently co-occupied by the ETS proteins ERG and FLI1 along with RUNX1 and GATA2 (Wilson et al. 2010). Our current study suggests that one or more KLF family members also contribute to the DNA binding specificity of TAL1. FLI1 and RUNX1 continued to cobind with TAL1 (along with GATA1 and GATA2) in megakaryocytes (Tijssen et al. 2011; Pimkin et al. 2014). Thus, binding of the ETS proteins and RUNX1 help direct TAL1 to regulatory sites in multipotential progenitor cells and in megakaryocytes (Fig. 8). In contrast, ETS proteins and RUNX1 rarely co-occupied TAL1 OSs in erythroid cells, and in fact the amount of co-occupancy declined with greater erythroid differentiation. These results are consistent with the critical role of RUNX1 in hematopoiesis (Okuda et al. 1996; Dowdy et al. 2010) and its down-regulation in erythroid cells (Lorsbach et al. 2004; North et al. 2004). Other TFs that cobind frequently with TAL1 in erythroid cells are members of the pentameric complex. In addition, KLF1 cobinds with TAL1 and GATA1 at a small number of key erythroid regulatory modules (Fig. 8; Tallack et al. 2010, 2012).

The major role played by GATA factors and ETS proteins in directing binding of TAL1:TCF3 to specific sites is reminiscent of observations on the binding of the TFs SMAD and TCF, which are the targets of the BMP and Wnt signaling pathways, respectively. These two TFs co-occupy DNA segments bound by lineage master regulators such as GATA1 and CEBPA, thereby directing SMAD and TCF to target genes (Trompouki et al. 2011). In a similar way, our data support a model of TAL1 being directed to a distinctive set of binding sites by cell type–specific combinations of TFs (Fig. 8).

The redistribution of TAL1 binding profoundly affects gene expression during hematopoietic differentiation, leading to dramatic changes in cell morphology and function. The new genomic sites to which TAL1 binds with each wave of redistribution place TAL1 in positions to regulate new sets of target genes. The cobinding master regulatory TFs not only direct TAL1 binding to the appropriate locations, but they also help to determine the impact of TAL1 on the target genes. In the cases illustrated in Figure 8, the impact is usually positive, i.e., associated with gene induction. Enhancement is associated with cobinding of TAL1 with a GATA factor in all three cell types: TAL1 with GATA2 in progenitor cells (Jing et al. 2008; Wozniak et al. 2008; Wilson et al. 2010), TAL1 with GATA1 and FLI1 in megakaryocytes (Wang et al. 2002; Tijssen et al. 2011; Dore et al. 2012; Pimkin et al. 2014), and TAL1 with GATA1 and sometimes KLF1 in erythroid cells (Cheng et al. 2009; Tripic et al. 2009; Soler et al. 2010; Tallack et al. 2010; Wu et al. 2011). The effects on gene expression are not exerted solely by the DNA-bound TFs, but rather by additional proteins and complexes recruited, including coactivators (Blobel et al. 1998) and regulators of chromatin-related processes such as BRD3 (Fig. 8; Lamonica et al. 2011). Conversely, TAL1 can play a role in repression of some genes by its association with co-repressors SIN3A, HDAC1 (Huang and Brandt 2000), or CBFA2T3 (also known as ETO2) (Schuh et al. 2005). Additional work is needed to discover the features that favor binding of TAL1 with proteins that promote either positive or negative regulation.

Our current study examined more closely particular aspects of the changes in regulation associated with the GATA switch, i.e., the replacement of GATA2 by GATA1 during maturation after erythroid commitment (Bresnick et al. 2010). We confirmed the previously described loss of TAL1 at many GATA switch sites and the repression of likely targets (Grass et al. 2006; Jing et al. 2008; Wozniak et al. 2008), but we also saw more frequently a retention of TAL1 after the GATA switch, which was associated with target gene induction. Another class of sites bound both by GATA2 and TAL1 simply lost TAL1 after the GATA switch, and this was associated with target gene repression. Thus the effects of the GATA switch are strongly correlated with the effect on TAL1 at bound sites. These results reemphasize the importance of cobinding of GATA1 and TAL1, presumably within the pentameric complex, in activating erythroid genes.

As is the case for almost all genome-wide mappings of TF occupancy, we found TAL1 occupying a very large number of genomic DNA segments. The 18,595 OSs in the combined data set were largely differentially bound in specific cell types, but each cell type still had thousands of bound segments. Consequently, each predicted target gene was associated with multiple TAL1 OSs, each of which was placed into categories based on binding across cell types or association with other TFs. These several multiplicities added complexity to the functional analysis of the predicted gene targets for the various categories of TAL1 OSs. We adopted two different methods for assigning gene targets to focus on the more robust results. It is important to realize that virtually every gene associated with TAL1 occupancy actually has several TAL1 OSs in its vicinity. Some of this multiplicity of binding likely reflects complex regulatory interactions that ensure correct timing and amount of expression, and some may reflect redundancy to achieve more robust regulation. Future work uncovering consistent trends in these networks of interactions should help illuminate the associated regulatory mechanisms. On the other hand, we cannot rule out the possibility that some of the binding sites are nonfunctional, e.g., they could be DNA segments with favorable motifs located in accessible chromatin, and TAL1 (or other TFs) not actively engaged in regulation could be bound there. Such “opportunistic” binding (John et al. 2011; Zhu et al. 2012) may explain some of the TAL1 OSs that show no obvious effect on presumptive target genes, such as those in categories with depletion (relative to all TAL1-associated genes) for both induction and repression (Fig. 6B). Future research, e.g., utilizing higher throughput experimental assessment of function and/or quantitative modeling of dynamic expression patterns based on genome-wide factor occupancy, should help answer the question of why there are so many binding sites.

The highly dynamic nature of TAL1 occupancy during hematopoiesis helps explain some apparent discrepancies in the literature. Studies with mouse erythroid progenitors (Kassouf et al. 2010) and in the G1E model system (Wu et al. 2011) led to the conclusion that TAL1 occupancy precedes that of GATA1, whereas studies of human CD36^+^ erythroid precursor cells led to a model of GATA1 binding before TAL1 (Hu et al. 2011). Clearly, TAL1 binds specifically to many genomic locations in multilineage precursor and erythroid progenitor cells, before GATA1 is produced abundantly. Once GATA1 is produced during erythroid differentiation, however, its binding appears to lead to a redistribution of TAL1 to locations containing GATA1.

## Methods

### TF occupancy data sets

TF occupancy was measured by chromatin immunoprecipitation (ChIP) followed by sequencing of the ChIP DNA on the Illumina Genome Analyzer IIx or HiSeq 2000 for a minimum of two biological replicates. ChIP was done for TAL1 in G1E cells, G1E-ER4 + E2 cells, Ter119^+^ erythroblasts, and megakaryocytes; for GATA1 in G1E-ER4 + E2 and Ter119^+^ erythroblasts; and for GATA2 in G1E and G1E-ER4 + E2, using antibodies sc-12984, sc-265, and sc-9008, respectively, from Santa Cruz Biotechnology (Wu et al. 2011; The Mouse ENCODE Consortium et al. 2014; Pimkin et al. 2014). The reads were mapped to mouse genome assembly mm9 using Bowtie (Langmead et al. 2009). Following the methods developed by the ENCODE Consortium (Landt et al. 2012), quality metrics were determined for each individual replicate and reproducibility was estimated by IDR (Li et al. 2011) or measures. Mapped reads from the replicates were pooled, and a single set of peaks was called by the program MACS (Zhang et al. 2008), with thresholds described in Wu et al. (2011) and Pimkin et al. (2014). The reads and peaks previously generated in our laboratory for TF binding sites are available at the NCBI Gene Expression Omnibus (GEO; http://www.ncbi.nlm.nih.gov/geo/) under accession number GSE30142. The peak sets and signal files of ChIP-seq data in HPC-7 and Ter119^−^ cells were downloaded from publications (Kassouf et al. 2010; Wilson et al. 2010) or from the UCSC Genome Browser (Kent et al. 2002). Certain genomic regions in mm9 were “blacklisted” because they had a high signal in the tracks for input DNA that was not from immunoprecipitated chromatin (Pimkin et al. 2014); peaks from any source falling in the blacklist regions were removed. An annotated list of all the TAL1 OSs is furnished as Supplemental Table 4.

### Transcriptome analysis in G1E and G1E-ER4 + E2 cells by RNA-seq

Total RNA was extracted from 5 million to 10 million G1E and G1E-ER4 (treated with estradiol for 30 h) cells using Invitrogen’s TRIzol reagent. Subsequent steps—including polyA selection, fragmentation, and cDNA synthesis—were performed as previously described (Mortazavi et al. 2008), with two changes to confer strand specificity (Parkhomchuk et al. 2009). Second-strand synthesis used dUTP rather than dTTP, followed by digestion of the uracil-containing, second-strand cDNA using uracil D-glycosylase during Illumina library preparation (prior to PCR amplification), thereby selectively amplifying first-strand cDNA. Libraries were prepared using the Illumina ChIP-seq kit and were sequenced on the Illumina HiSeq 2000 to obtain 2 × 99-nucleotide paired-end reads. All samples were determined as biological replicates. RNA-seq reads were mapped using TopHat2 in a reference-assisted manner (Trapnell et al. 2009; Kim et al. 2013; Paralkar et al. 2014). We used Cuffdiff2 (Trapnell et al. 2013) to identify DE genes, using the following options: dispersion-method = per-condition, library-type = fr-firststrand, max-bundle-frags = 20000000, min-reps-for-js-test = 2, −b for bias correction, and –M to mask globin transcripts. Transcript abundance levels pooled across replicates were expressed in terms of log_2_-transformed FPKMs (fragments per kilobase of exon model per million mapped fragments). Genes whose expression level exceeded our threshold for active expression (log_2_ FPKM > 3) in both cell types and whose differential expression passed a threshold of FDR = 0.05 were declared as DE.

### Comparison of TAL1 OSs among multiple cell types

The TAL1 OSs from the six cell types were concatenated and merged. The mean TAL1 ChIP-seq read counts were calculated for each segment in the merged set. We performed *k*-means, hierarchical, and model-based clustering on the quantile-normalized values. Heatmaps were generated with segments sorted by the clustering order. For better visualization, the signals that exceeded the 0.5% or 99.5% quantile in each cell type were forced to be the same color as the 0.5% or 99.5% quantile, respectively (Fig. 2A). Pearson correlation was calculated for each pairwise comparison of the raw ChIP-seq read counts between each cell type. The correlation matrix was converted into a dissimilarity matrix by subtracting the coefficients from one. A hierarchical clustering was performed on the dissimilarity matrix to give a dendrogram (Fig. 2B).

### Functional enrichment for presumptive target genes of TAL1 OSs

To find the genes potentially regulated by each TAL1 OS, we first found the EPU (Shen et al. 2012) that contained this OS and then identified all the genes whose TSSs were within the same EPU. We submitted the coordinates of the TSSs to the server for GREAT (McLean et al. 2010) to find the function-related terms enriched for the corresponding genes. We collected all the terms that GREAT found to be significantly associated with the potentially regulated gene sets for each category of TAL1 OSs. There are 11 gene sets based on the occupancy dynamics of their associated TAL1 OSs in the six cell types, plus one set that is not associated with any TAL1 OSs. Only the terms whose FDR *Q*-values passed 0.05 for both the binomial test and the hypergeometric test were examined further. In a second approach, the gene with the nearest TSS was assigned as the likely target gene for each TAL1 OS.

### Comparison of enrichment of five TF binding motifs on TAL1 OSs in five hematopoietic cell types

We used MEME-ChIP (Machanick and Bailey 2011) to find enrichment of known TF binding motifs in the TAL1 OSs of each cell type. Nucleotide sequences of the OSs were extracted through Galaxy (Goecks et al. 2010), and the sequences were masked by RepeatMasker (Smit and Green 1999) before being sent to MEME-ChIP. Locations of all occurrences of selected top ranking motifs were determined with FIMO (*P*-value set to 1 × 10^−3^). These selected motifs were also shown to be overrepresented in a comprehensive TF OS set from previous literature (Wilson et al. 2010). Binding site motifs (as position weight matrices, or PWMs) for GATA factors, the ETS factor SPI1 (PU.1), RUNX1, and KLF factors were downloaded from the JASPAR database (Sandelin et al. 2004). In order to find a strong TAL1 binding E-box motif, we first gathered the set of OSs that are occupied by TAL1 in G1E but not by either TAL1 or GATA1 in induced G1E-ER4, under the assumption that these should show enrichment for TAL1 binding directed primarily by its E-box motif, rather than indirect binding through GATA1 co-occupancy. Analysis of this set of OSs by the program DREME in the MEME-ChIP suite (Machanick and Bailey 2011) revealed the enriched E-box PWM that we used as the TAL1 binding motif. For each set of OSs, a histogram and a density curve were plotted to show frequency of motif locations versus the positions relative to the OSs centers. A dot plot was also used to show all the motif occurrences for each set of OSs that were sorted based on TAL1 occupancy strength (Ozdemir et al. 2011).

To quantitatively measure the motif enrichment in each panel, we first counted the number of motif occurrences within 50 bp on each side of the center of each OS (a) and in the remainder of the OS (b). Then we counted motif occurrences in comparably sized random DNA segments, again within 50 bp on each side of the center (c) and in the remainder of the OS (d). Enrichment (or depletion) was computed as log_2_ of the odds ratio of normalized counts in the center versus the remainder of the OS. Specifically, the odds ratio is [(average of a/average of c)/(average of b/average of d)].

### Gene expression responses associated with changes in TAL1 occupancy during GATA1-induced erythroid maturation

Co-location within an EPU was used to assign potential gene targets for each TAL1 OS. Each gene with a TSS in the same EPU as the TAL1 OS was assigned as a potential target. The expression response for each gene during maturation induced by restoration and activation of GATA1 in ER4 cells was determined from the RNA-seq data. Thus each TAL1 OS was associated with a specific number of genes that are induced, repressed, or not responding, and the percentage of DE genes that were induced or repressed was computed. Data for all the TAL1 OSs in each of the groups defined by overlaps in binding patterns (Fig. 6A) were combined by computing the mean of the percentages that were induced or repressed. To estimate a background frequency of induction or repression based on the TAL1 occupancy patterns, the TAL1 OS group designation (from Fig. 6A) was shuffled within the data matrix, and the (pseudo) average percentage of DE target genes that are induced and repressed was computed for each (pseudo) group. This shuffling and recomputation was repeated 1000 times. The mean of the (pseudo) averages gave the estimate of the background frequency of induction or repression, and these are shown as dotted lines in the boxplot of Figure 6B. We used the results for each of the shuffling and recomputations to produce a distribution of enrichment or depletion estimates. For each of the 1000 permutations, the true percentage of DE genes that were induced was divided by the percentage from the shuffled data set and likewise for the percentage of DE genes that were repressed. The log_2_ (true percentage/ percentage after shuffling) gives an empirical estimate of the enrichment or depletion for induction or repression of the presumptive gene targets in each TAL1 OS category. Since the permutations were done 1000 times, we could evaluate a distribution of these estimates for each TAL1 OS category.

## Data access

Data from this study were generated as part of the Mouse ENCODE Project (http://mouseencode.org/) and have been submitted to the NCBI Gene Expression Omnibus (GEO; http://www.ncbi.nlm.nih.gov/geo/) under accession numbers GSE36029, GSE36028, and GSE51338. Data are also available from the UCSC Genome Browser (tracks ENCODE/PSU on the mouse mm9 assembly) and a customized browser maintained at Penn State (http://main.genome-browser.bx.psu.edu).

## Supplementary Material

Supplemental Material
