# Supplementary material for: Dynamic shifts in occupancy by TAL1 are guided by GATA factors and drive large-scale reprogramming of gene expression during hematopoiesis
Source: Genome Res. 2014 Dec;24(12):1945–62. doi: 10.1101/gr.164830.113 (PMC4248312; doi:10.1101/gr.164830.113)
Supplement: Supplemental Material [file supp_24_12_1945__index.html]

Dynamic shifts in occupancy by TAL1 are guided by GATA factors and drive large-scale reprogramming of gene expression during hematopoiesis — Dynamic shifts in occupancy by TAL1 are guided by GATA factors and drive large-scale reprogramming of gene expression during hematopoiesis — Supplemental Material 

# Dynamic shifts in occupancy by TAL1 are guided by GATA factors and drive large-scale reprogramming of gene expression during hematopoiesis

## Supplemental Material

**Files in this Data Supplement:**

- Supplemental Material.docx
- Supplemental Table1.xlsx
- Supplemental Table2.xlsx
- Supplemental Table4.xlsx
