# Supplementary material for: Dynamic shifts in occupancy by TAL1 are guided by GATA factors and drive large-scale reprogramming of gene expression during hematopoiesis
Source: Genome Res. 2014 Dec;24(12):1945–62. doi: 10.1101/gr.164830.113 (PMC4248312; doi:10.1101/gr.164830.113)
Supplement: Supplemental Material [file supp_gr.164830.113_Supplemental_Material.docx]

**­Supplementary Material for**

**Dynamic shifts in occupancy by TAL1 are guided by GATA factors and drive large-scale reprogramming of gene expression during hematopoiesis**

Weisheng Wu ^1^, Christapher S. Morrissey ^1^, Cheryl A. Keller ^1^, Tejaswini Mishra ^1^, Maxim Pimkin ^2^, Nergiz Dogan ^1^, Gerd A. Blobel ^2,3^, Mitchell J. Weiss ^2,3^, Ross C. Hardison ^1^

^1^ Center for Comparative Genomics and Bioinformatics, Department of Biochemistry and Molecular Biology, The Pennsylvania State University, University Park, PA 16802

^2^ Division of Hematology, The Children’s Hospital of Philadelphia, Philadelphia, PA 19104, USA,

^3^ Perelman School of Medicine at the University of Pennsylvania, Philadelphia, PA 19104, USA

**Supplementary Table 1.** Enriched function terms for genes associated with TAL1 OSs in different occupancy patterns in the six cell types. The association is based on localization in the same EPUs. This is an Excel file furnished as a separate attachment. It can also be downloaded from:

http://bx.psu.edu/~weisheng/TAL1_manuscript/NewVersion/SuppTable1_byEPU_selectedgreatfunctionterms_significance_tal1ostypes_genes.xlsx

**Supplementary Table 2.** Enriched function terms for genes associated with TAL1 OSs in different occupancy patterns in the six cell types. The association is based on localization in the closest linear distance on the genome. This is an Excel file furnished as a separate attachment. It can also be downloaded from:

http://bx.psu.edu/~weisheng/TAL1_manuscript/NewVersion/SuppTable2_byProximity_selectedgreatfunctionterms_significance_tal1ostypes_genes.xlsx

**Supplementary Table 3.** Enriched motifs found by MEME-ChIP DREME on TAL1 OSs (only top ones are shown).

| HPC | 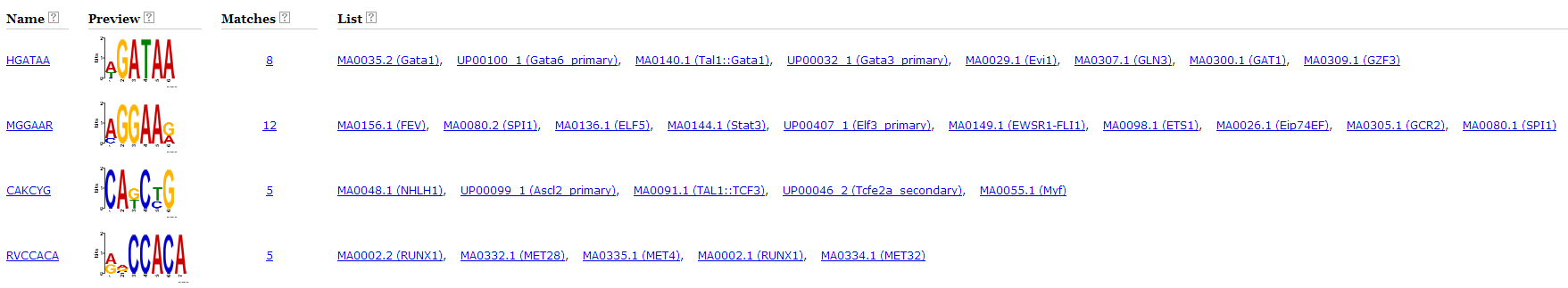 |
| --- | --- |
| EPro | 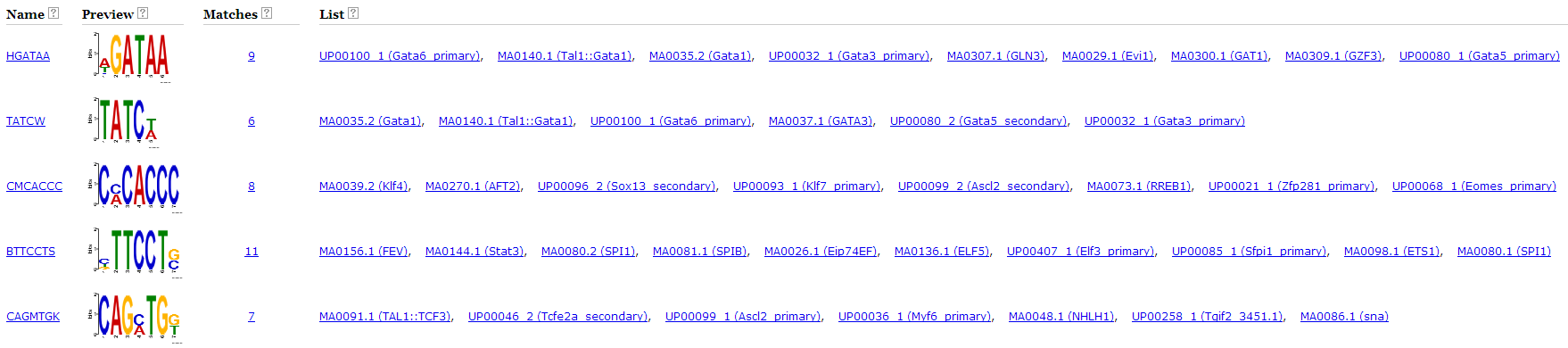 |
| G1E | 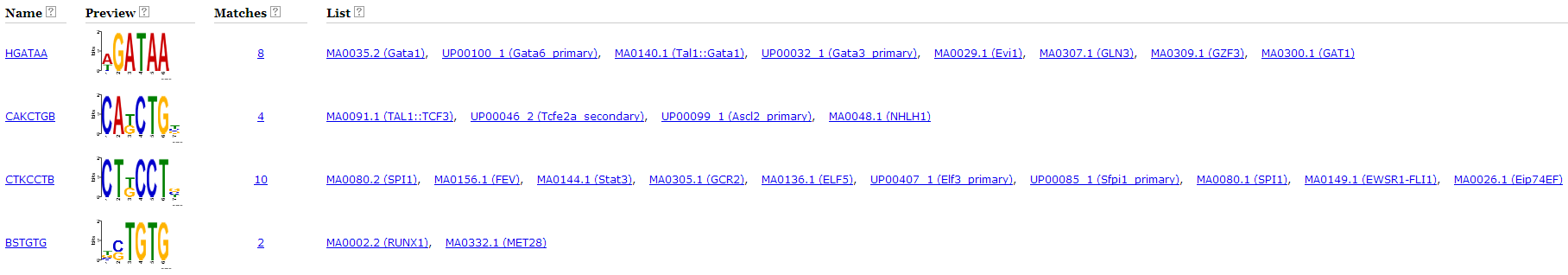 |
| ER4 | 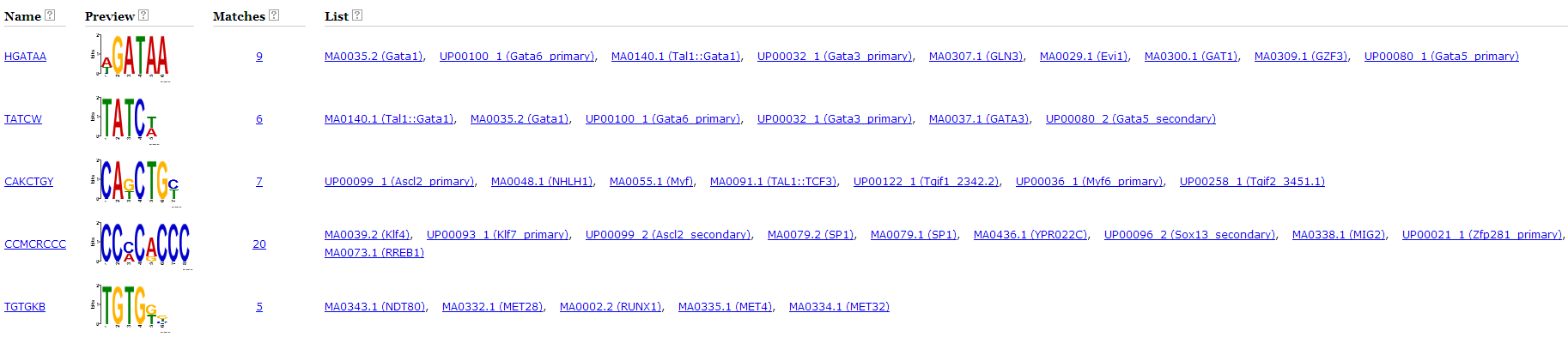 |
| Ebl | 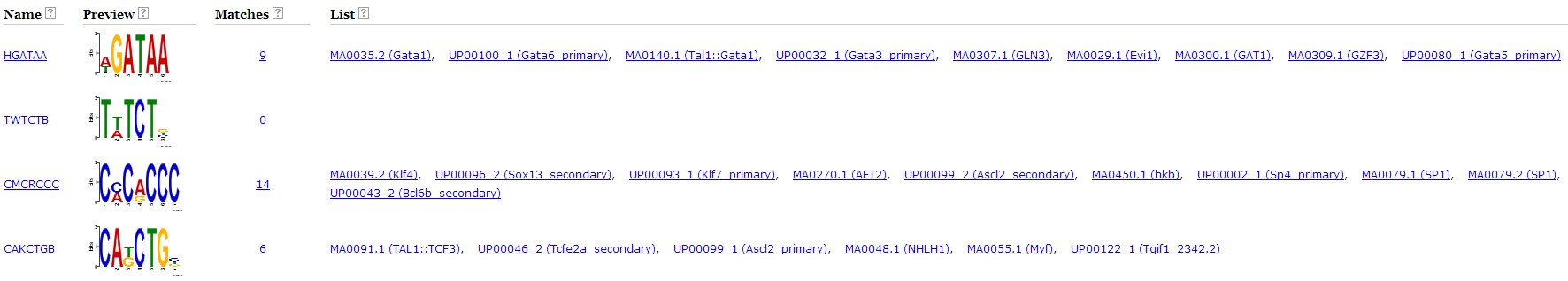 |
| Meg | 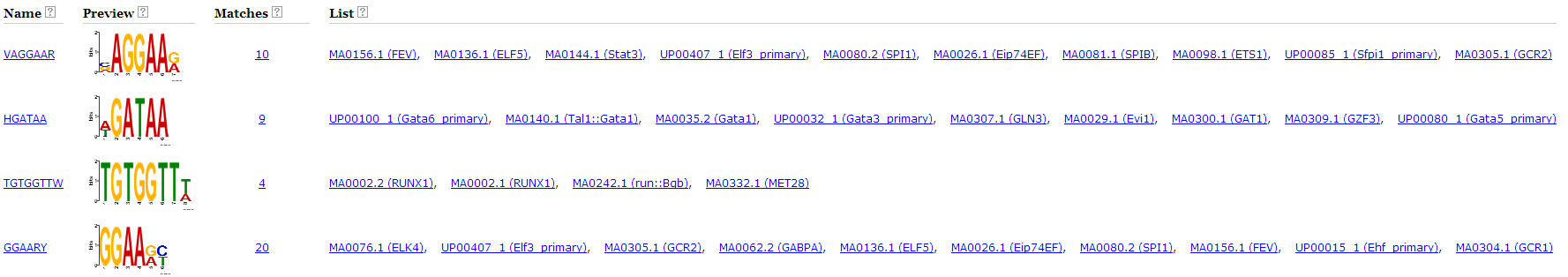 |

**Supplementary Table 4. TAL1 occupied segments in the different cell types.**

This is an Excel spreadsheet furnished as a separate file. It contains all the TAL1 OSs, with coordinates in mm9, and annotation about presence in each cell type and other features.


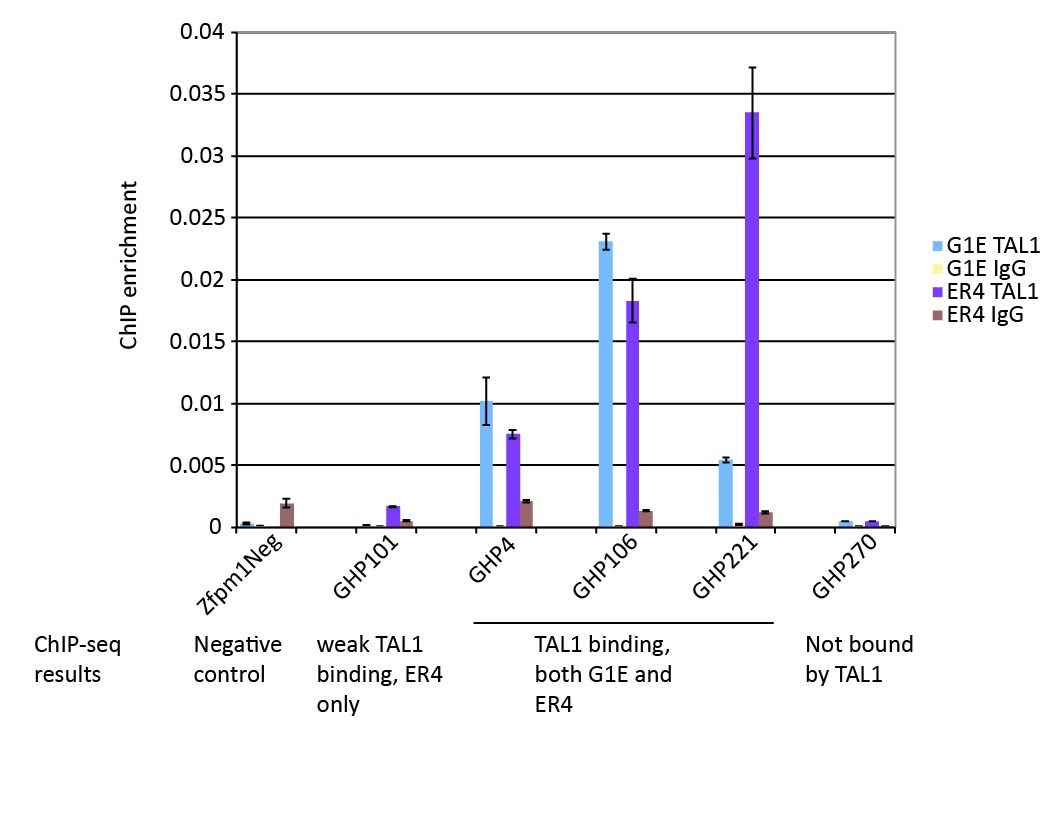


**Supplementary Figure 1. The occupancy patterns from ChIP-seq were confirmed at selected loci by ChIP-qPCR.** Using TAL1 ChIP DNA as template, quantitative PCR was performed on five GATA1 Hit Positive (GHP) sites and a negative control site. The qPCR results validated the peak-calling results from ChIP-seq data.





**Supplementary Figure 2. Hierarchical and model-based clustering of TAL1 OSs in the five cell types.** The normalized read counts on the TAL1 OS union set of peaks were clustered by (*A*) hierarchical clustering and (*B*) model-based clustering, shown by heatmaps.

**Supplementary Figure 3.** The examples of genes that carry lineage-specific TF occupancy, from the categories displayed in Table 2.

**Supplementary Figure 4.** Examples of genes that carry HPC7- and megakaryocyte-specific TAL1 occupancy, from the categories displayed in Table 2.

**Supplementary Figure 5.** The examples of genes that carry erythroid lineage-specific TAL1 occupancy, from the categories displayed in Table 2.

**Supplementary Figure 6.** The examples of genes that carry complex pattern of TF occupancy, from the categories displayed in Table 2.

**Negative regulation by TAL1 of genes predominantly expressed in other lineages**

In some cases, genes bound by TAL1 in one cell type (e.g. G1E) generated significant enrichment for functional terms associated with other cell types (e.g. MEG) (Table 2). We hypothesized that this could reflect, at least in part, negative regulation by TAL1 of the genes predominantly expressed in other lineages. To test this hypothesis, we extracted from the GREAT output the list of 61 genes bound by TAL1 in G1E that generated enrichment for the terms “abnormal megakaryocyte morphology” and “abnormal megakaryocyte progenitor cell morphology” in Table 2. We obtained their expression levels from the RNA-seq data in G1E cells and in MEGs, and computed the difference in levels as log2 (expression level in MEG/expression level in G1E). The histogram in Supplementary Figure 7 (below) shows that, of the genes whose expression is significantly different between the two cell types, many more are induced (25) in MEG than are repressed (9), relative to their expression in G1E. This result supports the hypothesis that the TAL1 occupancy in G1E for these MEG-associated genes is involved in negative regulation in G1E cells, at least in part.


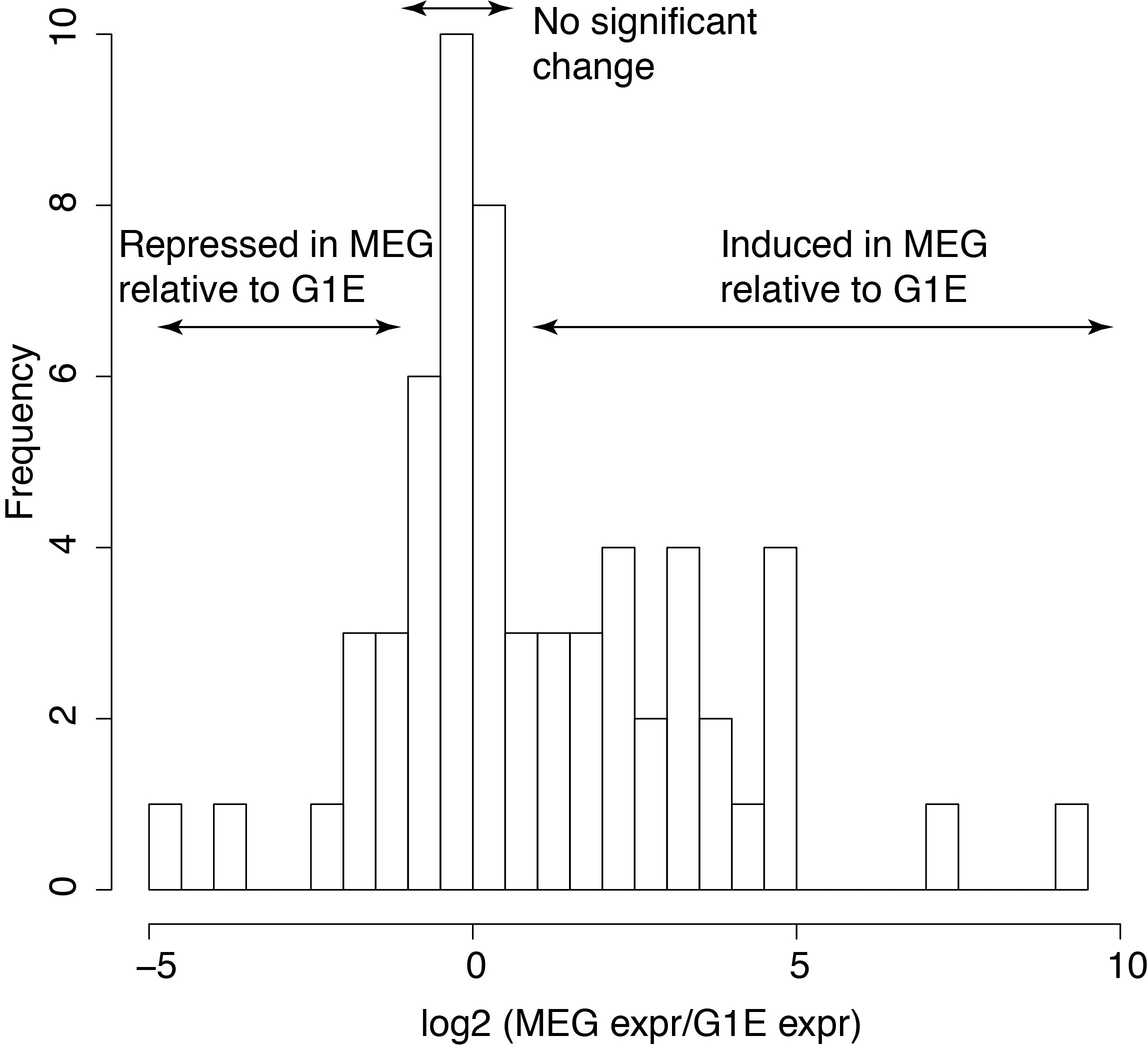


**Supplementary Figure 7.** Differences in expression level between MEG and G1E for genes bound by TAL1 in G1E cells that contribute for the MEG-associated terms in Table 2. The histogram shows the frequency at which genes with the designated expression differences were observed.


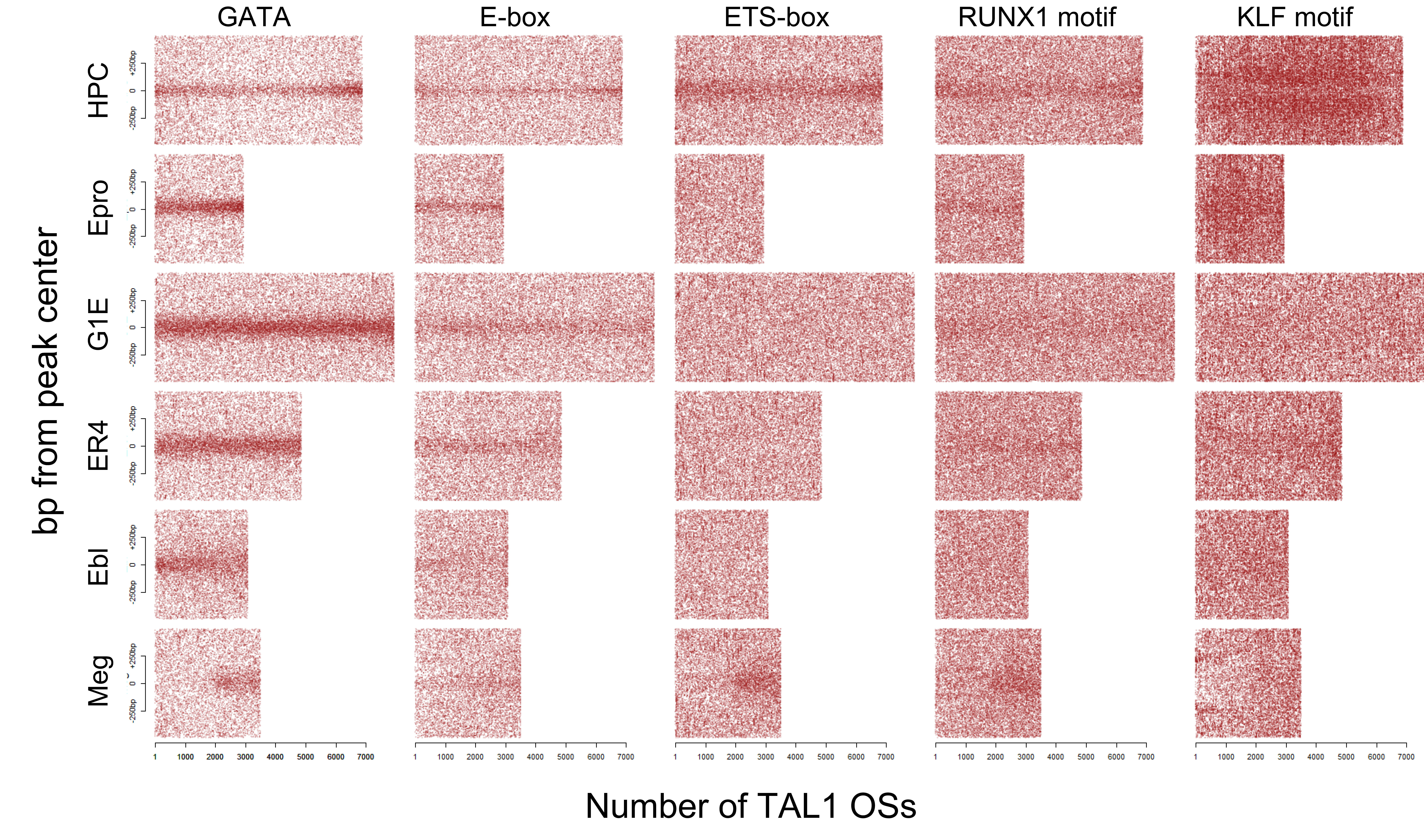


**Supplementary Figure 8.** **Distribution of five TF binding motifs on TAL1 OSs in the six cell types.** For each TAL1 OSs in the six cell types, the locations of all occurrences of motifs in 500bp upstream and downstream of OS centers were found by FIMO. These patterns are shown for each group of TAL1 OSs as a dot plot. In each plot, the TAL1 OSs were sorted by TAL1 binding signals increasing from left to right on the x-axis, and the location of each instance of a binding site motif was graphed along the y-axis for each TAL1 OS. The y-axis is centered on the peak of the binding signal for each TAL1 OS. The background occurrence of motif instances generates a diffuse pattern, whereas enrichment of motif instances in specific positions of the TAL1 OS generates a strong intensity.


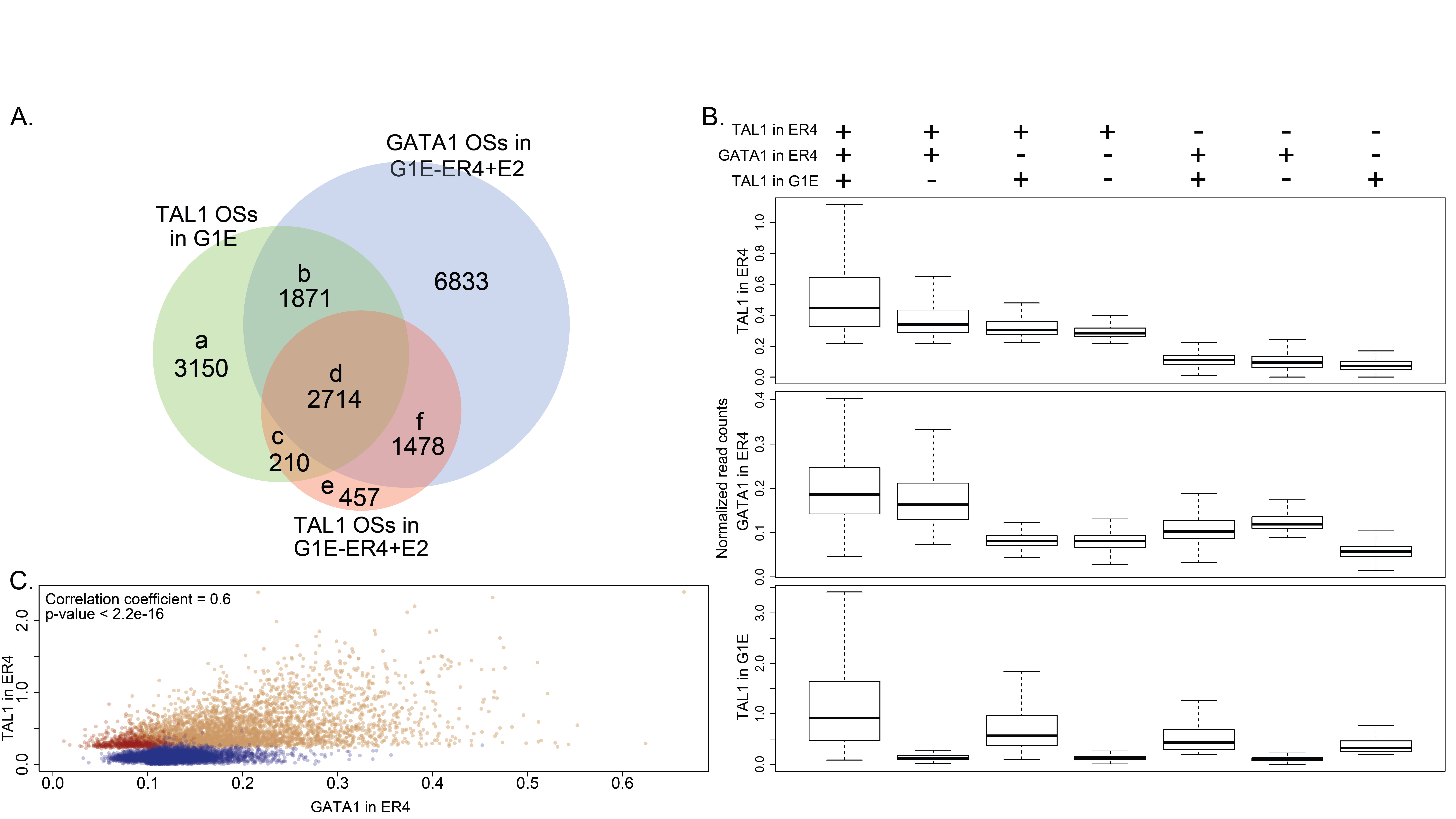


**Supplementary Figure 9.** **TAL1 occupancy and co-occupancy in the absence and presence of GATA1.** (*A*) Venn diagram showing the subset of TAL1 OSs in G1E and G1E-ER4+E2 cell lines that overlap with GATA1 OSs. (*B*) Normalized ChIP-seq read counts of TAL1 and GATA1 in G1E-ER4+E2 and TAL1 in G1E on the sets of DNA segments defined in (*A*). (*C*) Scatter plot showing the normalized ChIP-seq read counts of GATA1 and TAL1 read counts at GATA1 or TAL1 OSs in G1E-ER4+E2. The segments that are occupied only by TAL1, only by GATA1, or by both are represented by red, brown, or blue dots, respectively.


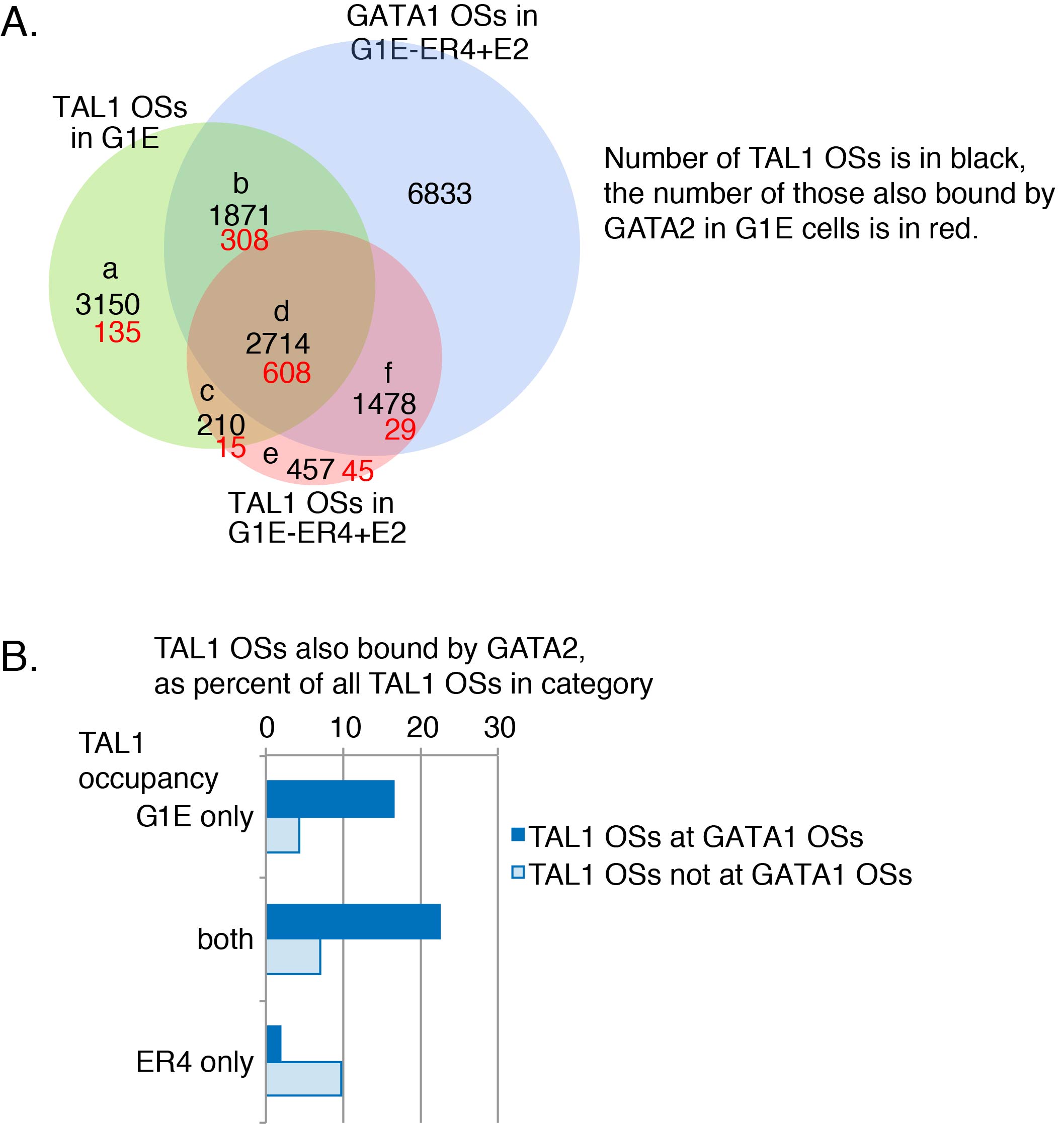


**Supplementary Figure 10. Role of GATA2 in the binding of TAL1 in G1E and ER4 cells.** The approximately 4000 DNA segments bound by GATA2, as ascertained in Wu et al. (2011, Genome Research 10:1659-1671), were intersected with the TAL1 OSs in G1E and ER4 cells. (A) The Venn diagram shows the numbers of TAL1 and GATA1 OSs (black numerals) in the categories determined by occupancy in G1E or ER4 cells; this is a repeat of Figure 6A. The number of TAL1 OSs in each category that also overlap with occupancy by GATA2 in G1E cells are shown as red numerals. (B) The proportion of the TAL1 OSs in each of the six categories that are also bound by GATA2 is plotted as a percentage. The occupancy categories are the same as groups a-f in panel A. The proportion relative to all the TAL1 OSs also bound by GATA2 are plotted in Fig. 6C in the main text.
